# Decidualized endometrial stromal cells present with altered androgen response in PCOS

**DOI:** 10.1038/s41598-021-95705-0

**Published:** 2021-08-11

**Authors:** Masuma Khatun, Alvin Meltsov, Darja Lavogina, Marina Loid, Keiu Kask, Riikka K. Arffman, Henna-Riikka Rossi, Freddy Lättekivi, Kersti Jääger, Kaarel Krjutškov, Ago Rinken, Andres Salumets, Terhi T. Piltonen

**Affiliations:** 1 Department of Obstetrics and Gynaecology, PEDEGO Research Unit, Medical Research Center, Oulu University Hospital, University of Oulu, Oulu, Finland; 2 Competence Centre on Health Technologies, Tartu, Estonia; 3 Department of Computer Science, University of Tartu, Tartu, Estonia; 4 Institute of Chemistry, University of Tartu, Tartu, Estonia; 5 Department of Obstetrics and Gynaecology, Institute of Clinical Medicine, University of Tartu, Tartu, Estonia; 6 Department of Pathophysiology, Institute of Biomedicine and Translational Medicine, University of Tartu, Tartu, Estonia; 7 Institute of Veterinary Medicine and Animal Sciences, Estonian University of Life Sciences, Tartu, Estonia; 8 Division of Obstetrics and Gynaecology, Department of Clinical Science, Intervention and Technology (CLINTEC), Karolinska Institutet, Stockholm, Sweden

**Keywords:** Cell biology, Molecular biology, Endocrinology, Medical research

## Abstract

Hyperandrogenic women with PCOS show disrupted decidualization (DE) and placentation. Dihydrotestosterone (DHT) is reported to enhance DE in non-PCOS endometrial stromal cells (eSC_Ctrl_); however, this has not been assessed in PCOS cells (eSC_PCOS_). Therefore, we studied the transcriptome profile of non-decidualized (non-DE) and DE eSCs from women with PCOS and Ctrl in response to short-term estradiol (E2) and/or progesterone (P4) exposure with/without (±) DHT. The non-DE eSCs were subjected to E2 ± DHT treatment, whereas the DE (0.5 mM 8-Br-cAMP, 96 h) eSCs were post-treated with E2 and P4 ± DHT, and RNA-sequenced. Validation was performed by immunofluorescence and immunohistochemistry. The results showed that, regardless of treatment, the PCOS and Ctrl samples clustered separately. The comparison of DE vs*.* non-DE eSC_PCOS_ without DHT revealed PCOS-specific differentially expressed genes (DEGs) involved in mitochondrial function and progesterone signaling. When further adding DHT, we detected altered responses for lysophosphatidic acid (LPA), inflammation, and androgen signaling**.** Overall, the results highlight an underlying defect in decidualized eSC_PCOS_, present with or without DHT exposure, and possibly linked to the altered pregnancy outcomes. We also report novel factors which elucidate the mechanisms of endometrial dysfunction in PCOS.

## Introduction

Polycystic ovary syndrome (PCOS) is the most common cause for anovulatory infertility. It involves endometrial alterations that are thought to contribute to reproductive performance^1^. The majority (60%) of women with PCOS exhibit hyperandrogenism, which is thought to be responsible for the pathogenesis of the syndrome^2^. This hyperandrogenism may also contribute to the increased risk for adverse pregnancy outcomes and placental aberrations^3^. Clinical findings have indicated that restoring ovulations with medication does not result in an endometrial environment comparable to that of ovulatory non-PCOS women, suggesting an underlying pathology^4^.

Endometrial decidualization involves dramatic morphological and functional changes coordinated by ovarian steroid hormones estrogen (E2) and progesterone (P4). Cyclic adenosine monophosphate (cAMP) activation provides synergistic enhancement of decidualization by inducing the transcriptome of common decidualization genes^5^. Supporting the crucial role of androgens in decidualization and endometrial receptivity, androgen receptor (AR)-knockout mice are reported to be sub-fertile, whereas upregulation of the AR in the endometrium results in impaired endometrial receptivity^6^. Progesterone has also been shown to down-regulate AR, modulating androgen effects in the endometrium^7^. Imbalance of this synergy may result in a disadvantageous environment for the implanting embryo, and subsequently a dysregulated placentation.

The PCOS endometrium has previously been shown to display altered expression of sex hormone receptors and their co-receptors, suggesting an increased E2 effect and a decreased P4 effect^8,9^. Additionally, studies suggest disrupted decidualization and placentation in hyperandrogenic mouse models of PCOS but also in hyperandrogenic women with PCOS^10,11^. While dihydrotestosterone (DHT; a more potent metabolite of testosterone) was reported to enhance decidualization in non-PCOS endometrial stromal cells (eSC_Ctrl_)^12^, this effect has not been observed in PCOS eSCs (eSC_PCOS_)^13^.

Based on these previous findings, the present study aims to evaluate the global gene expression profile of non-decidualized (non-DE) and decidualized (DE) eSCs in response to short-term steroid hormone exposure of PCOS compared to non-PCOS women. DHT was chosen over testosterone for the present study to bypass 5α-reductase dependence and to investigate isolated androgen effect with minimal testosterone to estradiol conversion^14,15^. The objective was to assess whether DHT differentially modulates non-DE eSCs when exposed to E2 (the model of non-decidualized/proliferative phase), compared to DE eSCs exposed to both E2 and P4 (the model of decidualized/mid-secretory phase) in PCOS and non-PCOS samples.

## Results

### Quantitative transcriptome profile after steroid hormone post-treatment in PCOS and Ctrl eSCs

**Figure 1:**
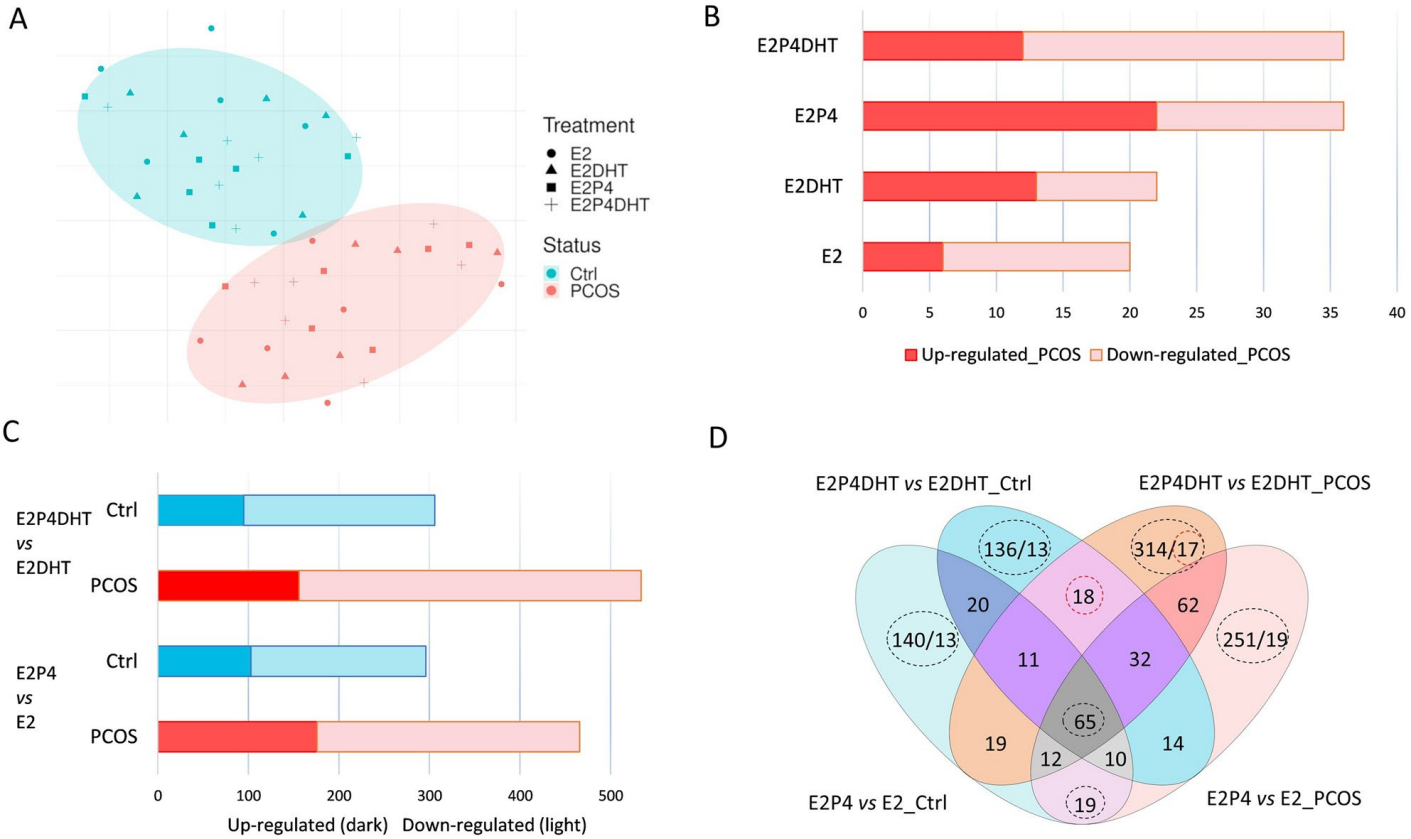
Quantitative transcriptome differences following steroid hormone exposure. (**A**) T-distributed stochastic neighbor embedding (t-SNE) plot of differentially expressed genes (DEGs) with FDR (False discovery rate) < 0.05 and absolute log_2_ fold change (LFC) > 2. Blue and red dots represent respectively endometrial stromal cells (eSCs) from Ctrl and PCOS. (**B**) Counts of up-regulated (red) and down-regulated (pink) DEGs in response to hormone treatment in PCOS group compared to Ctrl for non-decidualized (non-DE) eSCs post-treated with E2 with or without (±) DHT, and for decidualized (DE) eSCs post-treated with E2P4 ± DHT. (**C**) Counts of up- and down-regulated DEGs in Ctrl (blue) and PCOS (red) women in non-DE eSCs with E2 ± DHT and in DE eSCs with E2P4 ± DHT. (**D**) Venn diagram of group-wise comparisons illustrating the counts for the common vs. uniquely expressed DEGs for E2P4 vs. E2 and E2P4DHT vs*.* E2DHT in Ctrl (blue) and PCOS (red), respectively. E2, estrogen; P4, progesterone; DHT, dihydrotestosterone. The DEG counts, shown as ratios, illustrate the number of DEGs before and after applying Independent Hypothesis Weighting Bonferroni (IHW-BON) corrections for filtering the most significant hits. Validation targets were chosen from the DEG groups marked with a red circle.

**Figure 2:**
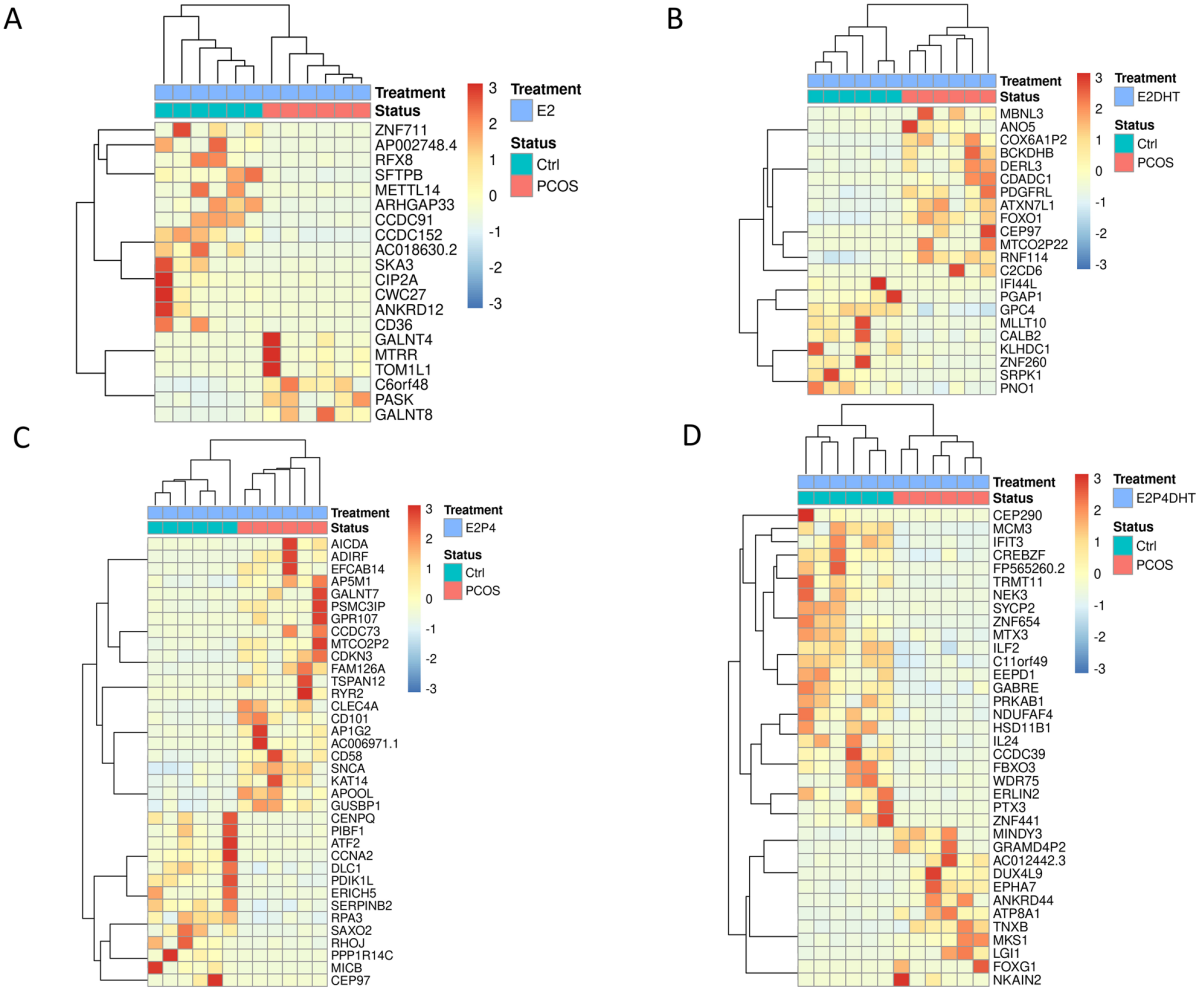
Self-organizing map (SOM) clustering analysis. Gene expression levels for Ctrl (cyan, n = 6) and PCOS (pink, n = 6) eSCs treated with (**A**) estrogen; E2 and (**B**) E2 with dihydrotestosterone; DHT (E2DHT) in non-decidualized (non-DE) eSCs; (**C**) E2 and progesterone; P4 without DHT (E2P4) and (**D**) E2 and P4 with DHT (E2P4DHT) treatment in decidualized (DE) eSCs. Red indicates high and blue indicates low expression level. Differentially expressed genes (DEGs) with false discovery rate (FDR < 0.05) were taken into consideration in the analyses.

Regardless of the treatment, the eSCs from PCOS and Ctrl women clustered separately in the t-SNE (T-distributed stochastic neighbor embedding) plot (Fig. 1A). In both non-DE and DE eSCs, as well as with and without DHT, there were 20–36 DEGs between PCOS vs. Ctrl groups. Most of the differences were observed in the DE-eSCs after E2P4 exposure (E2P4) (Fig. 1B), regardless of the DHT treatment. Based on SOM (Self-organizing map) analyses for each treatment, the PCOS and Ctrl groups also clustered separately (Fig. 2A–D). The non-DE eSC_PCOS_ post-treated with E2 without DHT featured differentially expressed genes (DEGs) related to cell signaling and metabolism (*GALNT4, TOM1L1, PASK, MTRR, ZNF711, CIP2A*), whereas E2 + DHT exposure triggered DEGs related to androgen action (*CDADC1, FOXO1, PDGFRL, KLHDC1, IFI44L*)*.* Following E2P4 post-treatment, DE-eSC_PCOS_ presented with DEGs related to cell cycle, proliferation, differentiation, and inflammation (E2P4: *CDKN3, CD58, SNCA, CCNA2, PIFB1, MICB*; E2P4DHT: *ILF2, NDUFAF4, GABRE, IFIT3, IL24, PTX3, NEK3*). In the presence of DHT (E2P4DHT vs*.* E2DHT), there was no major change in the number of DEGs in the eSC_Ctrl_, whereas in eSC_PCOS_, there was an increase in the number of DEGs (p = 0.029, Fig. 1C). A Venn diagram depicting the DEGs for comparison of E2P4 ± DHT vs. E2 ± DHT, is presented in Fig. 1D. A detailed listing of the DEGs can be found in Supplemental Table S1A–C.

### Common DEGs for decidualization with steroid post-treatment

In the Venn diagram (Fig. 1D), 65 genes were common for decidualization in all four group-wise comparisons regardless of PCOS status or hormone treatment. These genes were thus considered conserved decidualization genes of eSCs in vitro. An additional 19 genes were identified to be specific for E2P4 vs. E2 comparison, both in Ctrl and PCOS. The 65 conserved genes included commonly known decidualization genes such as *SST, REN, MMP3, POSTN, PSAT1,* and *COL1A2,* whereas the 19 DEGs included mostly genes related to cell cycle regulation: *SPDYE16*, *FLT3*, *HDKC1*, *ACTG2, N4BP2, IFIT2*, *SFRP4* and *ZWINT*. A total of 18 DEGs detected comparing E2P4DHT vs. E2DHT were common for both PCOS and Ctrl, including genes involved in cytoskeleton and/or cell migratory properties (*PLPPR4*, *LPAR1*), and cell differentiation or proliferation (*STC2, EZH2, HJURP, ANG, CENPN*). A detailed list of these DEGs is shown in the Supplemental Table S2 A–C. Lysophosphatidic Acid Receptor 1 (*LPAR1*), which showed reduced expression in DE eSCs after DHT exposure in both Ctrl and PCOS, was chosen for validation.

### Unique DEGs associated with steroid hormone post-treatment in decidualized vs. non-decidualized eSCs

The PCOS vs. Ctrl DEGs that were characteristic of the DE vs. non-DE comparison were assessed after steroid hormone post-treatment. This comparison revealed 140 unique group-specific DEGs for eSC_Ctrl_ and 251 for eSC_PCOS_ without DHT exposure, and revealed 136 DEGs for eSC_Ctrl_ and 314 for eSC_PCOS_ with DHT exposure (Fig. 1D, Supplemental Table S3A–D). After correction for multiple testing (IHW-BON), the remaining DEG count was 13/140 in Ctrl and 19/251 in PCOS for the E2P4 vs. E2 comparison in the absence of DHT (Supplemental Table S4A, B). The 13 unique genes for eSC_Ctrl_ included genes involved in cellular function (*SKA3, GATA6, SAXO2*), immune response (*GPANK1, STK17B*), and ion channel function (*KCNK2, KCNB1*). The 19 unique DEGs for the eSC_PCOS_ were mostly related to fatty acid and mitochondrial energy metabolism (*RARRES1, EGR2*, *FABP5, SLC2A3, CKMT1B, ABCB1/MDR1*) and progesterone signaling (*HHIP*). The IHW-BON corrected DEGs (13/136) for E2P4DHT vs*.* E2DHT in eSC_Ctrl_ included genes predominantly related to immune response (*CXCL8/IL-8*, *ATG4C, IL-24*, *MMP10*, *TNFSF4*)*.* The same comparison in eSC_PCOS_ (17/314) included DEGs related to cellular calcium phosphate homeostasis and intracellular trafficking (*PHEX*, *PGAP1, RAB3C, SNX1, C2CD6*)*,* inflammation (*GBP1, RCN3, BBX*), and androgen action related cell stemness (*KDM7A, IFI27, ALDH1A1, C2CD6*) (Supplemental Table S4C, D). From these DEGs, Aldehyde Dehydrogenase 1 Family Member A1 (*ALDH1A1*), which was downregulated in DE eSC_PCOS_ after DHT exposure, was chosen as a validation target.

### Functional enrichment analysis of DEGs

According to the Reactome and GO annotation assignment, the pathways related to the 65 common DEGs for decidualization (E2P4 ± DHT) were significantly (FDR < 0.05) enriched for processes such as cell proliferation, collagen formation, extracellular matrix modulation, and the cell cycle. The only significant pathway for the 18 common DEGs in the E2P4DHT vs. E2DHT comparison was related to the lysosphingolipid and the lysophosphatidic acid (LPA) receptor. For the 314 PCOS group-specific DEGs arising from the E2P4DHT vs. E2DHT comparison, pathways related to the cell cycle, interferon signaling, endosomal pathway, nucleosome assembly, and immunoregulatory interactions were found to be enriched. Detailed data are shown in Supplemental Table S5A–G.

### In vitro and in vivo LPAR1 and ALDH1A1 protein validation

**Figure 3:**
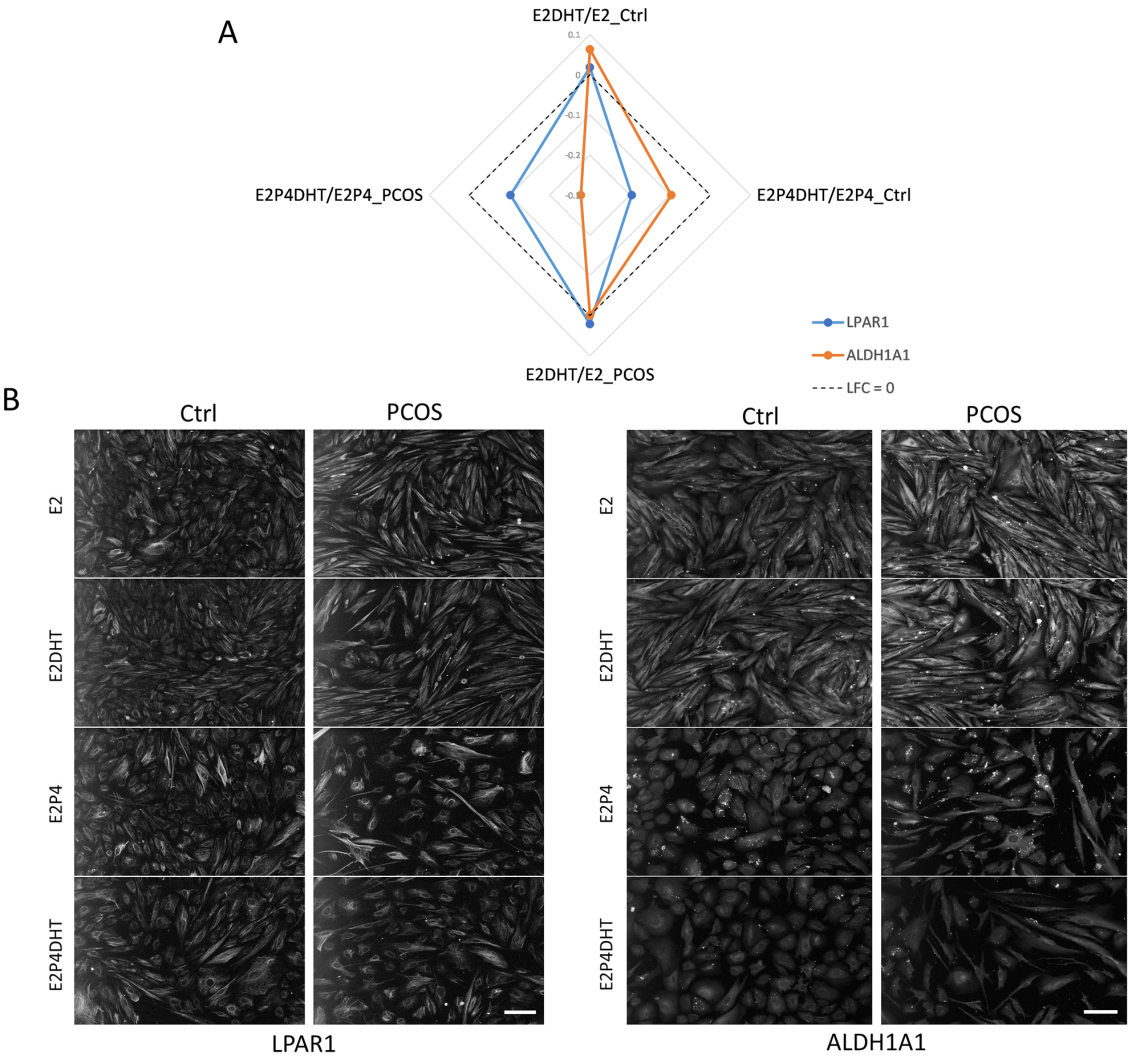
Validation of in vitro protein expression by immunofluorescence (IF). (**A**) Radar plot showing in vitro effect of DHT on expression of LPAR1 (blue) or ALDH1A1 (orange) in non-DE eSCs with E2 ± DHT and in DE-eSCs with E2P4 ± DHT in both groups (Ctrl, PCOS; both n = 3). The y-axis shows log fold change (LFC) values calculated for ratios with and without DHT; LFC 0 line (no change in expression) is shown in dotted black. (**B**) IF panel for LPAR1 and ALDH1A1 protein expression in non-DE-eSCs with E2 or E2DHT post-treatment and DE-eSCs with E2P4 or E2P4DHT post-treatments in eSC_Ctrl_ and eSC_PCOS_; nuclei are stained with DAPI. Scale bar: 100 µm.

**Figure 4:**
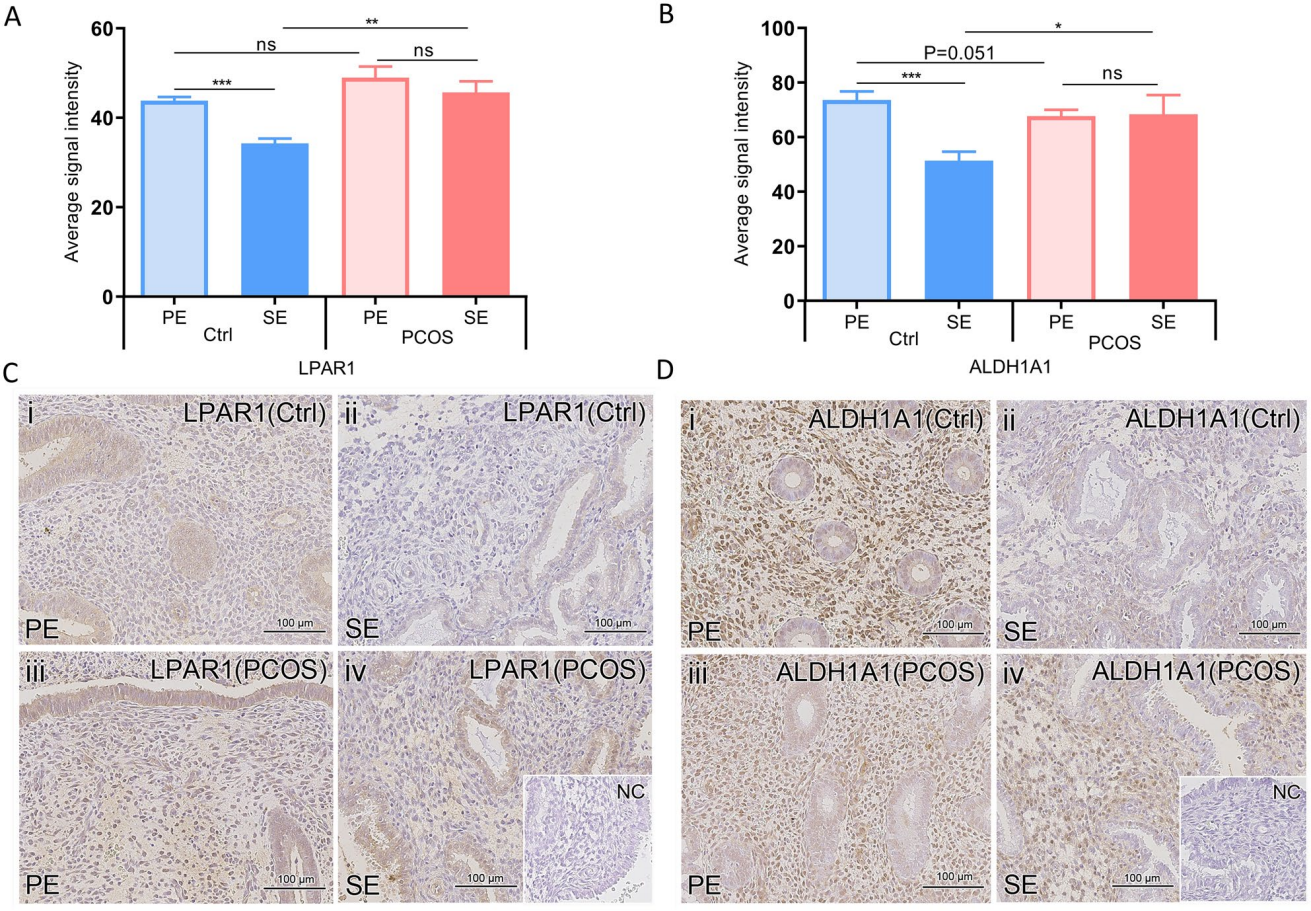
Validation of in vivo protein expression by immunohistochemistry (IHC). (**A**, **B**) In vivo protein expression showing LPAR1 and ALDH1A1 expression in the endometrial stroma in Ctrl and PCOS; n = 6, proliferative endometrium (PE): cycle day (6–9); Ctrl, PCOS; n = 5, secretory endometrium (SE): collected 9–10 days after the luteinizing hormone surge. (**C**, **D**) Whole biopsy samples from PE and SE in Ctrl and PCOS were used for IHC staining to identify LPAR1 and ALDH1A1 proteins. For every patient, 3 different areas (700 × 700 µm) on each slide were considered and stroma was separated from the glandular epithelium manually for staining quantification. Scale bar 100 µm; NC; negative control.

For validation of the RNA-seq data, two candidate genes were chosen representing two distinct groups of DEGs in the Venn diagram (Fig. 1D; red circles): *LPAR1* (a DE-down-regulated gene in E2P4DHT vs. E2DHT that was common to both groups) and *ALDH1A1* (a DE-down-regulated gene E2P4DHT vs. E2DHT that was unique to PCOS). The IF data for the in vitro decidualized eSCs post-treated with steroid hormones is presented in Fig. 3. According to the logarithmic fold change (LFC) ratio based on E2 and E2P4 treatments ± DHT, down-regulation of both LPAR1 and ALDH1A1 could be observed in DE eSC_PCOS_ and eSC_Ctrl_ post-treated with DHT. Consistent with the RNA-seq data, ALDH1A1 showed more pronounced downregulation in eSC_PCOS_ compared to eSC_Ctrl_ (Fig. 3A, B). The IHC analysis of all endometrium biopsy samples showed significant downregulation of LPAR1 and ALDH1A1 in the stromal compartment in SE vs*.* PE comparison only in the Ctrl group (p < 0.001), whereas no significant change was detected in PCOS samples (Fig. 4A–D).

## Discussion

This is the first study assessing a global gene expression profile after a short-term steroid hormone exposure in vitro in eSC_PCOS_ compared to eSC_Ctrl_. Our specific interest was to investigate the effect of DHT in the presence of E2 (modeling PE) or E2P4 (modeling SE) in non-DE and DE eSCs, respectively. We hypothesized that the hormonal challenges may reveal an underlying defect in the eSC_PCOS_, which could be linked to the altered placentation and adverse pregnancy outcomes reported in vivo.

The clear clustering of the RNA-seq study samples in PCOS and Ctrl group both in the t-SNE plot and the SOM suggests a distinct transcriptome profile for the eSC_PCOS_, which includes many previously unreported genes, especially for the PCOS endometrium (Figs. 1A, B, 2). It is known that the PCOS endometrium is burdened with an adverse metabolic environment arising from factors such as visceral obesity, insulin resistance, and hyperinsulinemia, all common for the syndrome^4^. In line with this, our study was also able to identify up-regulation of genes in the non-DE eSC_PCOS_ with E2 post-treatment, which have been previously reported to be involved in lipid and glucose metabolism (*GALNT4, GALNT8, PASK*)^16,17^. On the other hand, the E2DHT post-treatment in eSC_PCOS_ revealed several DEGs related to androgen action (*CDADC1, FOXO1, PDGFRL*), supporting the previous findings that estrogen promotes androgen action in the non-PCOS and PCOS endometrium^18–20^. Even though the eSC_PCOS_ showed comparable decidualization capacity with the eSC_Ctrl_ by classical decidualization markers *PRL* and *IGFBP-1*, the global gene expression profile after short-term hormonal exposure in DE-eSC_PCOS_ was different from the one in DE-eSC_Ctrl_. Several of the increased (*SNCA, CDKN3, CD58*) or decreased (*PIBF1, MICB*) DEGs have been previously described in endometriosis, endometrial cancer, and other gynecological pathologies as being specifically involved in delayed decidualization and inflammation^21–24^. Moreover, the addition of DHT to E2P4 in DE eSCs resulted in a complete change in gene expression in eSC_PCOS_, including modulation of many progesterone receptor (PR) target genes such as *IFIT3, IL24, PTX3*, and *NEK3*, all of which are involved in cell proliferation, decidualization, and inflammation. Interestingly, these genes have also been reported to be associated with endometriosis and implantation failure^25–28^. Collectively, these findings support the impaired action of P4 in the PCOS endometrium in a hyperandrogenic milieu, in line with previous studies^4^.

Decidualization, a well-coordinated transformation process of eSCs from an E2-dominant proliferative state to an E2P4-driven differentiated state, is imperative for successful embryo attachment, implantation, and healthy pregnancy. The central role of decidualization also necessitates identifying the set of robust conservative genes that drive this important process. Accordingly, we identified 65 DEGs (Fig. 1D) common for decidualization, regardless of the study groups and DHT exposure. Of these 65 DE-consensus genes, 10 were identical and 6 were homologous to genes that have been included in the endometrial receptivity array (ERA)^29^. There were also 19 DEGs that were common for decidualization in the absence of DHT (E2P4 vs. E2) and were found in both groups; these are involved in the cell cycle and metabolic processes (*HKDC1, ACTG2, ZWINT, IFIT2*) and are crucial for ensuring the normal endometrial milieu^30,31^. In addition, 19 unique DEGs (Supplemental Table S4B) were identified for the E2P4 vs. E2 comparison in eSC_PCOS_ without added DHT, underlining the distinct transcriptome profile for DE-eSC_PCOS_. Among the upregulated DEGs in this PCOS/DE-group, *RARRES1* is involved in androgen signaling via retinoic acid synthesis^32^, and *EGR2* and *FABP5* are both involved in fatty acid uptake^33,34^. In the same DEG group, the downregulated transporter genes are associated with mitochondrial function (*CKMT1B, ABCB1/MDR1*)^35,36^. In addition, a well-known mediator of the progesterone signaling cascade *HHIP* was found to be downregulated, implying an impaired decidualization process for eSC_PCOS_^37,38^. This is consistent with our previously reported data, which indicated a robust impairment of decidualization in more severe PCOS cases^39^. The milder phenotype of the PCOS cases included in the present study may explain why the underlined impairment was not seen in classical decidualization markers but in the larger gene panel after steroid hormone exposure. The concept of progesterone resistance and mitochondrial dysfunction in the PCOS endometrium is also supported by a recent work in PCOS-mimicking animals, where DHT exposure during pregnancy resulted in impairment of the endometrial progesterone effect, decidualization, and mitochondrial function^40,41^.

Previous studies have reported that normal levels of androgens modulate the P4 effect, thereby facilitating decidualization and normal endometrial function^42^. The comparison of E2P4 vs. E2 in the presence of DHT (E2P4DHT vs. E2DHT) yielded 18 DEGs (Fig. 1D) common for both study groups, representing DHT-dependent decidualization genes. As expected, these DEGs were involved in androgen receptor (AR) regulation (*PLPPR4, STC2, ANG, LPAR1*), suggesting the interference of DHT with lysophosphatidic acid (LPA) signaling, which is involved in a variety of cellular processes such as proliferation, differentiation, adhesion, and tissue morphology^43–45^. Recently, the molecular influence of the LPA receptor on implantation was discovered, as its targeted deletion in mice resulted in significantly reduced litter size, which could be attributed to delayed implantation and altered embryo development^46^. However, the suppression of LPAR1 is apparent in the secretory DE stromal cells in control/fertile women, as seen in our study (Figs. 3, 4). These changes were not as uniform in PCOS women and may thus contribute to the endometrial dysfunction in PCOS. Thus, our study offers a unique and intriguing link between DHT effects on eSCs decidualization and altered phospholipid signaling. Future studies will be required to clarify whether this connection has a role in PCOS-related endometrial dysfunction.

In a PCOS mouse model, the DHT exposure has also been shown to induce impaired decidualization and implantation that could be linked to poor vasculature, angiogenesis, and placental formation in these animals^47^. In our study, the 17 DEGs unique to PCOS in the E2P4DHT vs*.* E2DHT comparison (Supplemental Table S4D), have been previously reported to be upregulated in cases with thin endometrium and implantation failure for example (*PHEX, RNASE7, SNX1*)^48^. Among the down-regulated DEGs in the same group, there were several genes connected to androgen-related cellular signaling (*KDM7A, ALDH1A1, C2CD6*)^49,50^, as well as genes important for implantation (*GBP1, RCN3, BBX, IFI27*)^51–54^. Of these, ALDH1A1 was chosen for validation as it has been shown to be specific for androgen action and plays a role in several physiological processes such as lipid and glucose metabolism^55^. In DE eSC_PCOS_, *ALDH1A1* was downregulated after DHT exposure in RNA-seq analysis, suggesting a desynchronized androgen effect in PCOS women compared to non-PCOS Ctrl. In vitro validation by IF for this protein was in line with our RNA-seq data. Although in vivo IHC analysis confirmed down-regulation of ALDH1A1 during decidualization in non-PCOS Ctrl women, the trend was not evident in tissue samples derived from PCOS women in IHC studies. This apparent contradiction can be easily explained by the fact that intact tissue samples from the natural cycle do not explicitly mirror the RNA-seq results performed on isolated chemically induced DE eSCs in vitro.

Unlike eSC_PCOS_, the 13 DEGs in eSC_Ctrl_ (E2P4DHT vs. E2DHT comparison, Supplemental Table S4C) featured several inflammatory cascade-related genes suggesting that the eSC_Ctrl_ are highly stressed during androgen exposure. Considering all the differences found between the eSCs from fertile/Ctrl and PCOS women, we suggest that the eSCs from non-PCOS women should not be used to model PCOS, but rather primary cells from PCOS subjects should be used due to an inherently altered steroid hormone response in the PCOS endometrium. This change was also evident in the absence of DHT, and especially in the case of decidualized eSCs in vitro*,* as they presented with progesterone resistance and mitochondrial dysfunction. DHT was able to cause other changes unique to eSC_PCOS_. This likely implies altered epigenetic regulation in eSC_PCOS_^56^, although yet requires further research.

The strengths of the study include a well-defined sample set and experimental design including exposure with three central steroid hormones in different combinations and comparisons between cases and controls. Regarding limitations, the in vitro decidualization model and limited sample size is not well comparable with the in vivo system. In addition, the 24 h hormone exposure after decidualization can be considered short, although short-term exposure was already able to reveal unique differences between study groups. Further replication of the findings and functional validation in a larger sample set should be pursued in future studies.

## Conclusion

The data herein provides, for the first time, insight into decidualized eSC_PCOS_ and their steroid hormone response using global gene expression profiling. The results highlight an underlying defect in decidualized eSC_PCOS_ that is present with or without DHT exposure. This may suggest the presence of cellular memory, likely involving the epigenetic mechanisms. These changes may provide functional insights into endometrial dysfunction in women with PCOS and pave the way for future studies on biological themes such as progesterone resistance, LPA signaling, and mitochondrial dysfunction. The results of the present study may also give insight into the altered endometrium milieu for embryo implantation and adverse pregnancy outcomes in PCOS-affected women. Further studies are also warranted to establish whether women with different PCOS phenotypes have different risks and mechanisms for endometrial dysfunction.

## Materials and methods

### Patient characteristics and sample collection

Endometrial biopsies were collected from women that were diagnosed with PCOS at the Oulu University Hospital (According to the Rotterdam criteria and the new PCOS guideline^57,58^) and from non-PCOS women (Ctrl). Exclusion criteria included pregnancy or breastfeeding within 6 months of collection and hormonal or anti-diabetic medication within 3 months of collection. The dating of the collected endometrial tissue samples utilized in all the experiments was evaluated by an experienced pathologist by examining the tissue morphology with hematoxylin and eosin (H/E) staining, and corresponded to the respective cycle phase. The study was approved by the Ethical Committee of Northern Ostrobothnia Hospital District. Informed consent was obtained from all participants in accordance with the Declaration of Helsinki.

### Isolation of endometrial stromal cells (eSCs), in vitro decidualization, and hormone exposure

Endometrial biopsy samples were collected at the proliferative phase (PE) of the menstrual cycle (cycle days [cd] 6–10) from Ctrl (mean age/body mass index (BMI) ± standard error of the mean (SEM); age 35 ± 2.3 years, BMI 27 ± 0.5 kg/m^2^) and from women with PCOS (age 35 ± 1.0 years, BMI 27 ± 2.6 kg/m^2^). Endometrial stromal cells were isolated from these biopsies as previously described^39^. There were no significant differences between the groups in age or BMI.

**Figure 5:**
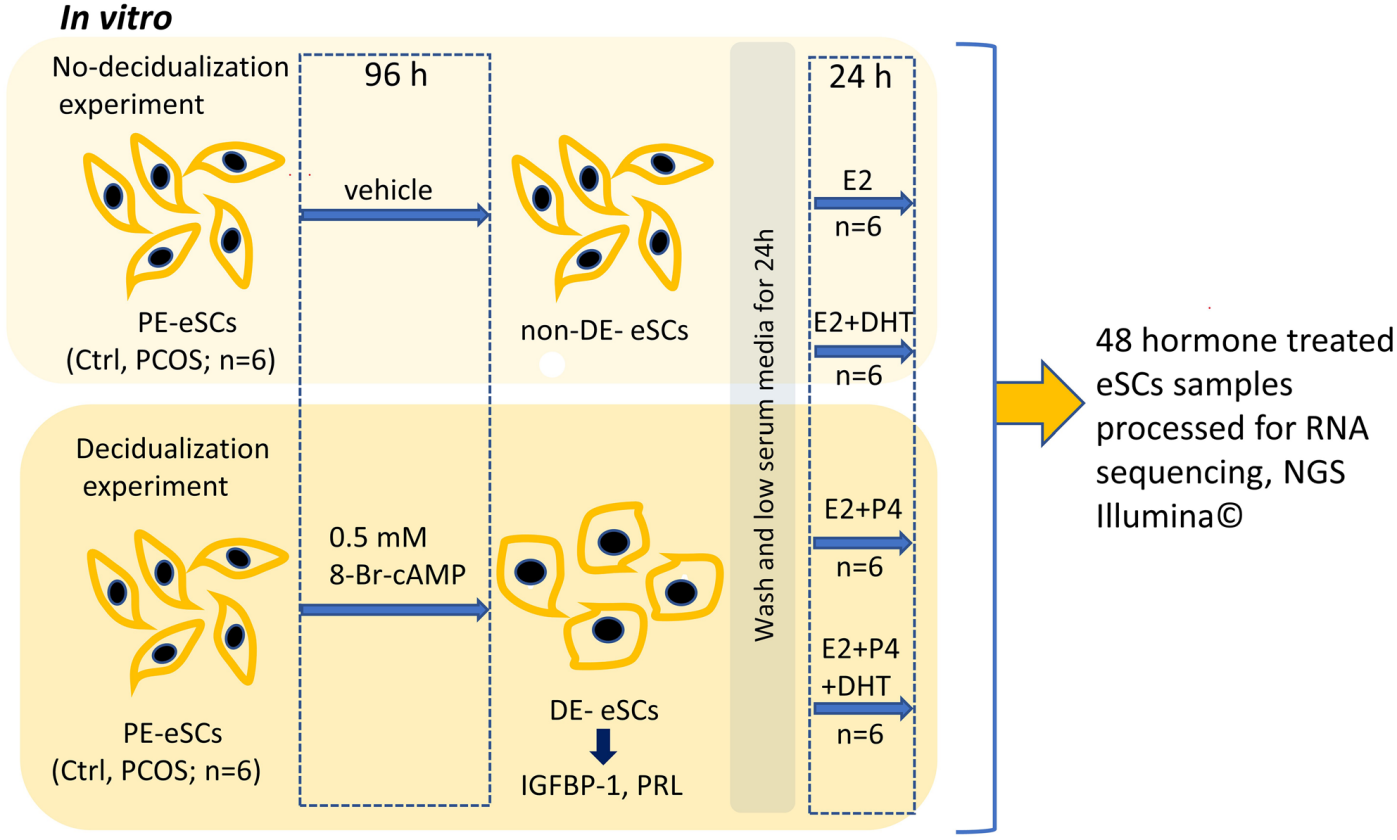
Schematic diagram of workflow. Proliferative phase (PE, cycle day 6–9) endometrial stromal cells (eSCs) from women with PCOS and non-PCOS controls (both n = 6) were decidualized (DE; 0.5 mM 8-Br-cAMP; stock solution prepared in DMSO) and non-decidualized (non-DE; 0.1% DMSO, vehicle) for 96 h. Cells were washed and starved in low serum media for 24 h. Non-DE eSCs were subjected to post-treatment with estrogen alone (E2, 10 nM) or with dihydrotestosterone (DHT, 100 nM; E2 + DHT) for 24 h. The DE-eSCs were subjected to post-treatment with E2 and progesterone (P4, 1 µM; E2 + P4) or E2P4 with DHT (100 nM; E2 + P4 + DHT) for 24 h. All four-hormone combination treated cells were harvested, and RNA sequencing (NextSeq500, Illumina) was performed from all different treatments’ arms (n = 48).

For decidualization (DE), eSCs from Ctrl and PCOS (eSC_Ctrl_, eSC_PCOS_, n_total_ = 12) were treated with 8-Br-cAMP (0.5 mM, Sigma-Aldrich, Germany) in duplicate for 96 h in a low serum medium^5^, while non-decidualized (non-DE) cells were cultured for 96 h using only DMSO (vehicle; Sigma-Aldrich, Germany). Cells were than washed and starved for 24 h in low serum media. Next, eSCs were post-treated for 24 h with hormones in low serum medium: non-DE eSCs were treated with E2 (10 nM, Sigma-Aldrich, Germany) with or without (±) DHT (100 nM, Sigma-Aldrich, Germany)^59^; while the DE eSCs were post-treated with E2 (10 nM, Sigma-Aldrich, Germany) in combination with P4 (1 µM, Sigma-Aldrich, Germany) (E2P4) ± DHT. Decidualization was confirmed by analyzing the increased expression of prolactin (PRL) and insulin-like growth factor-1 (IGFBP-1) both in individual Ctrl and PCOS. There were no significant differences between the groups (Supplemental Figure 1). The detailed study protocol is presented in Fig. 5.

### RNA sequencing, data processing, and bioinformatic analysis

Forty-eight samples in duplicate (total number of 96 samples) from four different hormone combination treatments as well as both groups were used for RNA isolation and cDNA library preparation. Total RNA was extracted using RNeasy Mini Kits (Qiagen, Valencia, USA) and RNA sequencing (RNA-seq) libraries were prepared using the SMART-seq2 protocol with modifications^60^. Initial data retrieval and processing is outlined in the Supplemental Methods Part 1. The RNA-seq data are available in the GEO database (accession: GSE171507). The differential expression and subsequent bioinformatics analysis are presented in Supplemental Methods Part 2.

### Reverse transcription quantitative polymerase chain reaction (RT-qPCR)

**Figure 6:**
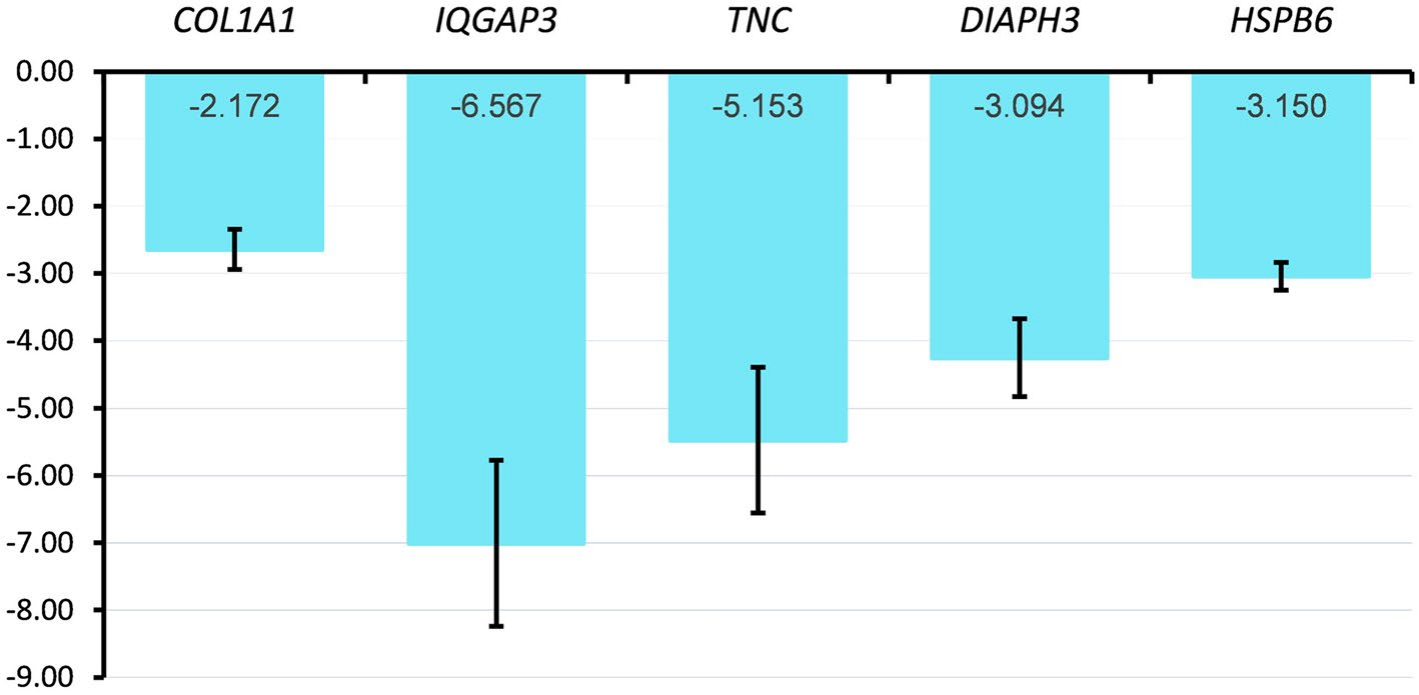
Technical validation of RNA-seq by reverse transcription quantitative polymerase chain reaction (RT-qPCR). Differentially expressed genes (DEGs) with false discovery rate, FDR < 0.05 were chosen from E2P4DHT vs*.* E2DHT comparison (eSC_Ctrl_, n = 3). The log_2_ fold change (LFC) for RT-qPCR is shown on the y-axis as the blue bar. The numerical LFC value retrieved from RNA-seq data is specific for the treatment corresponding gene shown inside each bar. Data are presented as mean ± standard error of the mean (SEM). *TBP* and *GAPDH* were used as reference genes.

For technical validation of sequencing data, RT-qPCR was performed according to the protocol described in Supplemental Methods Part 3. The results are shown in Fig. 6. The oligonucleotide primers are presented in Supplemental Table S6.

### In vitro studies and immunofluorescence (IF) for LPAR1 and ALDH1A1 proteins

Proliferative phase (cd 6–10) eSCs from controls (n = 3, age 35 ± 2.1 years, BMI 28 ± 3.5 kg/m^2^) and women with PCOS (n = 3, age 31 ± 2.0 years, BMI 26 ± 2.8 kg/m^2^) were treated with 8-Br-cAMP or 0.1% DMSO (DE vs*.* non-DE) for 96 h, then treated with hormones for 24 h as described earlier. The cells were fixed with methanol, and then stained with primary antibodies (anti-LPAR1 and anti-ALDH1A1, 1:100) and a nuclear stain (DAPI). The detailed protocol is described in Supplemental Methods Part 4.

### In vivo studies and immunohistochemistry (IHC) for LPAR1 and ALDH1A1 proteins

The PE phase endometrial biopsies were obtained in (cd 6–10); (Ctrl, n = 6, age 35 ± 2.3 years, BMI 27 ± 0.5 kg/m^2^; PCOS, n = 6, age 35 ± 1.0 years, BMI 27 ± 2.6 kg/m^2^). The SE phase endometrial biopsies were also obtained 9–10 days after a surge in luteinizing hormone (LH) confirmed by the Clearblue Advanced Digital Ovulation Test kit and by a pathologist based on endometrial histology (Ctrl, n = 5, age 28 ± 3.3 years, BMI 24 ± 2.2 kg/m^2^; PCOS, n = 5, age 33 ± 2.4 years, BMI 25 ± 0.6 kg/m^2^). Formalin-fixed paraffin-embedded sections were used for IHC analysis using primary antibodies (anti-LPAR1, 1:400 and anti-ALDH1A1, 1:1000). The detailed protocol is described in Supplemental Methods Part 5.

## Supplementary Information

Supplementary Methods.

Supplementary Table S1.

Supplementary Table S2.

Supplementary Table S3.

Supplementary Table S4.

Supplementary Table S5.

Supplementary Table S6.

Supplementary Figure 1.

Supplementary Captions.
