# Supplementary material for: Decidualized endometrial stromal cells present with altered androgen response in PCOS
Source: Sci Rep. 2021 Aug 11;11:16287. doi: 10.1038/s41598-021-95705-0 (PMC8357821; doi:10.1038/s41598-021-95705-0)
Supplement: Supplementary file 1 — Supplementary Methods. [file 41598_2021_95705_MOESM1_ESM.docx]

**Supplementary Methods**

***Part 1. Library preparation, Next Generation Sequencing (NGS) and data retrieval***

Instead of single cells and original template-switching oligonucleotide (TSO), 12 ng of total RNA for cDNA synthesis and 1 μM TSO (biotin-AAGCAGTGGTATCAACGCAGAGTrGrGrG, Sigma, Schnelldorf, Germany) in combination with 200 nM anchored oligo-T primer (biotin-TTAAGCAGTGGTATCAACGCAGAGTCGACT30+V, where + is LNA nucleotide, Exiqon, Vedbaek, Denmark) was used. Ten cycles of PCR with Phusion High-Fidelity DNA Polymerase (Thermo Scientific, HL, Finland) compatible with the original protocol were used for pre-amplification. 5 µl of cDNA was applied to single-index library preparation using Nextera XT DNA Sample Preparation Kit (Illumina, FC-131-1024, San Diego, CA, USA). AMPure XP beads (Beckman Coulter, San Jose, CA, USA) were used for all clean-up steps. The quality of the resulting libraries was assessed with the 2200 TapeStation system (Agilent, Edinburg, UK). Libraries were pooled and sequenced on NextSeq500 using High Output Flow Cell v2.5 (single-end, 80 bp; Illumina).

After acquiring sequencing data using Illumina BaseSpace, the reads were quantified with Salmon 0.9.1 ^1^ in quasi-mapping mode using indexed Ensemble v95 annotation. The quality control (Q30) of raw sequencing data and statistical summary on aligned counts was performed with FastQC 0.11.5 ^2^ and MultiQC 1.7 ^3^. Based on QC, further data transformation was performed by trimming the adapter size of 57 nucleotides with Trimmomatic 0.38 ^4^. The quantified transcript read counts were summarized to genes using Bioconductor packages tximport 1.10.1 ^5^.

***Part 2. Differential expression and subsequent bioinformatics analysis***

Differential expression analysis was performed using DESeq2 1.22.2 ^6^ and p-values were adjusted for multiple testing using the Benjamini-Hochberg procedure. Results were considered statistically significant if they had a false discovery rate (FDR) <0.05 for all downstream analysis. Data manipulation for visualization was performed using R (3.5.1) packages kohonen 3.0.8; Self Organizing Map (SOM) ^7^; T-Distributed Stochastic Neighbor Embedding; Rtsne 0.15 ^8^; pheatmap 1.0.12 ^9^ and venny2.1.0 ^10^. For further functional analysis, differentially expressed genes (DEGs) were filtered out using the Independent Hypothesis Weighted Bonferroni correction (IHW-BON <0.05) ^11^. Pathway over-representation analysis of the selected gene sets was conducted using the clusterProfiler ^12^ and ReactomePA ^13^ packages.

***Part 3. Reverse transcription quantitative polymerase chain reaction (RT-qPCR)***

For technical validation of sequencing data, RT-qPCR was carried in triplicate as described earlier ^14^ on selected DEGs (*IQGAP3, HSPB6, TNC, COL1A1,* and *DIAPH3* with E2P4DHT *vs.* E2DHT treatment in eSC_Ctrl_, n=3).

Data were analyzed using 7500 Software v2.0.5 (Applied Biosystems), and differences in gene expression levels were estimated using the comparative Ct (2^-ΔΔCt^) method ^15^. Wilcoxon paired-sample test was performed to observe the significance level.

***Part 4. Protocol used for the in vitro studies and immunofluorescence (IF)***

**Chemicals and antibodies**

The solutions and growth medium components for the cell culture were obtained from the following sources: phosphate-buffered saline (PBS), charcoal-purified fetal bovine serum (ccFBS), L-glutamine and Dulbecco's Modified Eagle’s medium (DMEM) / Ham’s F12 medium without phenol red – Sigma-Aldrich (Steinheim, Germany); a mixture of penicillin, streptomycin, and amphotericin B – Capricorn (Ebsdorfergrund, Germany). The chemicals used for the *in vitro* decidualization and hormonal treatment [8-bromoadenosine 3′,5′-cyclic monophosphate sodium salt (8-Br-cAMP), oestradiol (E2), progesterone (P4), 5α-dihydrotestosterone (DHT)] were from Sigma-Aldrich (Steinheim, Germany). The stock solutions of compounds (1000×) in cell culture grade DMSO (AppliChem; Darmstadt, Germany) were stored at -20 °C; DHT was stored at -90 °C.

For microscopy, the cells were grown on 24-well Ibidi black m-plates (Ibidi GmbH, Gräfelfing, Germany). For fixation of cells, methanol was obtained from Honeywell (Riedel-de Haën™, Seelze, Germany). For preparation of washing and blocking solutions in IF experiments, Triton X-100 from AppliChem (Darmstadt, Germany), bovine serum albumin fraction V (BSA) from Capricorn Scientific (Ebsdorfergrund, Germany) and PBS (supplemented with Ca^2+^, Mg^2+^) from Sigma-Aldrich (Steinheim, Germany) were used. Primary antibodies were obtained from the following sources: anti-LPAR1, rabbit polyclonal IgG against human lysophosphatidic acid receptor (code SAB4500689) – Sigma-Aldrich (Saint Louis, Missouri, USA); anti-ALDH1A1, mouse monoclonal IgG against human aldehyde dehydrogenase 1 family member (code sc-374149) – Santa Cruz Biotechnology (Dallas, Texas, United States). Secondary antibodies [goat cross-adsorbed antibody against rabbit IgG (H+L), conjugated with Alexa Fluor® 568; goat cross-adsorbed antibody against mouse IgG (H+L), conjugated with Alexa Fluor® 647] and the nuclear stain 4',6-diamidino-2-phenylindole (DAPI) were from Invitrogen (Eugene, Oregon, USA).

**Treatment of cells**

Endometrial stromal cells (eSCs; Ctrl, PCOS, n=3) were pre-grown on 10 cm Petri dishes for 2-3 passages in phenol red-free DMEM/Ham’s F12 supplemented with 10% ccFBS, L-Gln and a mixture of penicillin, streptomycin, and amphotericin B at 37 °C in 5% CO_2_ incubator. Upon reaching confluency, half of the cells from the Petri dish were suspended in the growth medium and seeded onto one 24-well microscopy plate. After 24hrs, the growth medium was replaced with the starvation medium (2% ccFBS instead of 10% ccFBS), which was used until the end of experiment. Following 24hrs starvation, 96hrs treatment of cells with 0.5 mM 8-Br-cAMP or 0.1% DMSO (vehicle) was started. Subsequently, the cells were treated with hormones (24hrs): 10 nM E2 (E2) or E2 + 100 nM DHT (E2DHT) in case of cells pre-treated with DMSO; and E2 + 100 nM P4 (E2P4) or E2 + P4 + DHT (E2P4DHT) in case of cells pre-treated with 8-Br-cAMP. On each plate, two duplicate wells were used for the identical treatments.

**Immunofluorescence (IF) and microscopy**

Following hormonal treatment of eSCs, the medium was removed, and the cells were rinsed with PBS and fixed with cold methanol (15 min at -20 °C). Next, methanol was removed, and the cells were washed twice with PBS. Subsequently, blocking with 1% BSA in PBS (w/v) was carried out for 1 h at room temperature (RT), followed by the overnight incubation with primary antibodies at 4 °C. The following dilutions (in 1% BSA/PBS) of antibodies were used: anti-LPAR1, anti-ALDH1A1, 1:100. On the next day, fixed cells were washed three times with 0.1% solution of Triton X-100 in PBS, and 3-6 h incubation with secondary antibodies (1:1000 dilution in 1% BSA/PBS) at 4 °C was carried out. The washing procedure was repeated, followed by staining with 300 nM solution of DAPI in PBS. Finally, the cells were washed twice with PBS and stored in PBS at 4 °C until imaging.

Fluorescence microscopy was carried out with Cytation 5 multi-mode reader (BioTek; Winooski, VT, USA) using 10x air objective. The following LED and filter cubes were used: for DAPI, 365 nm LED and DAPI filter block; for Alexa Fluor® 568, 523 nm LED and RFP filter block; for Alexa Fluor® 647, 623 nm LED and Cy5 filter block. Two images per well were taken, one at the center of the well and one at a different randomly chosen location. The imaging settings (LED intensity/signal integration time/detector gain) were as follows: for nuclear stain, 5/110/15; for cells stained with anti-LPAR1 9/515/19; for cells stained with anti-ALDH1A1, 5/169/19.

**Data analysis**

For general data analysis, GraphPad Prism 6 (San Diego, CA, USA) and Excel 2016 (Microsoft Office 365; Redmond, WA, USA) were used. For microscopy data quantification, ImageJ software (Fiji package) was used ^16^. The signal intensity in each individual channel was measured using raw grayscale images by drawing a straight line and quantifying mean intensity in the intracellular location characteristic of the given antibody (anti-LPAR1: along filaments; anti-ALDH1A1: cytoplasmic). Such quantification was carried out for 12 randomly chosen cells per field of view. The data collected for the same antibody, treatment and cell type from 2 fields of view and 2 different wells (a total of 48 cells) was pooled for each independent experiment. Next, for each antibody, logarithm of fold change (LFC2) was calculated for the averaged signals for the differently treated cells from the same patient: E2DHT ratio to E2 and E2P4DHT ratio to E2P4 (DHT-based LFC). Finally, the LFC values of the same type (DHT-based) obtained in three independent experiments were pooled for each antibody and treatment separately for both groups and the averaged LFC2 values were plotted onto the wind rose plot.

***Part 5. Protocol used for Immunohistochemistry (IHC)***

For IHC, Formalin-fixed paraffin-embedded the tissue sections (3 μm, 2 sections per slide) on slides were deparaffinized and rehydrated according to standard protocol (Abcam IHC guide). A mouse and rabbit specific HRP/DAB (ABC) Detection IHC Kit (ab64264, Abcam, UK) was used. All steps were carried out according to the producer’s protocol, except antigen retrieval pre-treatment, which was not used. Primary antibody dilutions (in 1% BSA/PBS) of antibodies were used: anti-LPAR1 – 1:400, and anti-ALDH1A1 – 1:1 000. Primary antibodies were obtained from the following sources: anti-LPAR1, rabbit polyclonal IgG against human lysophosphatidic acid receptor (code SAB4500689) – Sigma-Aldrich (Saint Louis, Missouri, USA); anti-ALDH1A1, mouse monoclonal IgG against human aldehyde dehydrogenase 1 family member (code sc-374149) – Santa Cruz Biotechnology (Dallas, Texas, United States). Incubation with primary antibody was carried out 1 hour in a humidity chamber at room temperature. Further steps were carried out as per manufacturer’s protocol. Chromogenic reaction was developed for 30 seconds and stopped thereafter. Cell nuclei were counterstained with Mayer´s haematoxylin solution for 15 seconds, then the slides were washed for 10 minutes with tap water. Slides were dehydrated through graded ethanol and xylene solutions and mounted with Leica CV mount (Leica Biosystems, US).

**Image and data analysis**

Slides were scanned using Leica SCN 400 Slide Scanner (Leica Biosystems, US) with maximum of 20x magnification objective. Semi-quantitative analysis of three different areas of scanned sections was conducted with ImageJ package Fiji version 1.52e ^16^. Briefly, the intensity of DAB signal was measured for stromal components of the endometrium. The relative DAB intensity was calculated using the formula: f = 255 - i, where “f” is relative DAB intensity and “i” is mean DAB intensity obtained from the software ranging from 0 (zero – deep brown, highest expression), to 255 (total white) ^17^. The Wilcoxon -Mann-Whitney test was performed to determine statistical significance. GraphPad Prism 6 (San Diego, CA, USA) and ImageJ software (Fiji package) were used for data quantification and analysis.

References

1. Patro, R., Duggal, G., Love, M. I., Irizarry, R. A. & Kingsford, C. Salmon provides fast and bias-aware quantification of transcript expression. *Nat. Methods* **14**, 417-419 (2017).

2. Wingett, S. W. & Andrews, S. FastQ Screen: A tool for multi-genome mapping and quality control. *F1000Res* **7**, 1338 (2018).

3. Ewels, P., Magnusson, M., Lundin, S. & Kaller, M. MultiQC: summarize analysis results for multiple tools and samples in a single report. *Bioinformatics* **32**, 3047-3048 (2016).

4. Bolger, A. M., Lohse, M. & Usadel, B. Trimmomatic: a flexible trimmer for Illumina sequence data. *Bioinformatics* **30**, 2114-2120 (2014).

5. Soneson, C., Love, M. I. & Robinson, M. D. Differential analyses for RNA-seq: transcript-level estimates improve gene-level inferences. *F1000Res* **4**, 1521 (2015).

6. Love, M. I., Huber, W. & Anders, S. Moderated estimation of fold change and dispersion for RNA-seq data with DESeq2. *Genome Biol.* **15**, 550-014-0550-8 (2014).

7. Wehrens, R., Kruisselbrink,J. Flexible Self-Organizing Maps in kohonen 3.0. *Journal of Statistical Software* **87** (2018).

8. Maaten, L. J. P. V. Accelerating t-SNE using Tree-Based Algorithms. *Journal of Machine Learning Research* **15**, 3221-3245 (2014).

11. Ignatiadis, N., Klaus, B., Zaugg, J. B. & Huber, W. Data-driven hypothesis weighting increases detection power in genome-scale multiple testing. *Nat. Methods* **13**, 577-580 (2016).

12. Yu, G., Wang, L. G., Han, Y. & He, Q. Y. clusterProfiler: an R package for comparing biological themes among gene clusters. *OMICS* **16**, 284-287 (2012).

13. Yu, G. & He, Q. Y. ReactomePA: an R/Bioconductor package for reactome pathway analysis and visualization. *Mol. Biosyst* **12**, 477-479 (2016).

14. Suhorutshenko, M. *et al*. Endometrial receptivity revisited: endometrial transcriptome adjusted for tissue cellular heterogeneity. *Hum. Reprod.* **33**, 2074-2086 (2018).

15. Livak, K. J. & Schmittgen, T. D. Analysis of relative gene expression data using real-time quantitative PCR and the 2(-Delta Delta C(T)) Method. *Methods* **25**, 402-408 (2001).

16. Schindelin, J. *et al*. Fiji: an open-source platform for biological-image analysis. *Nat. Methods* **9**, 676-682 (2012).

17. Fuhrich, D. G., Lessey, B. A. & Savaris, R. F. Comparison of HSCORE assessment of endometrial beta3 integrin subunit expression with digital HSCORE using computerized image analysis (ImageJ). *Anal. Quant. Cytopathol. Histpathol* **35**, 210-216 (2013).
