# Supplementary material for: Decidualized endometrial stromal cells present with altered androgen response in PCOS
Source: Sci Rep. 2021 Aug 11;11:16287. doi: 10.1038/s41598-021-95705-0 (PMC8357821; doi:10.1038/s41598-021-95705-0)
Supplement: Supplementary file 3 — Supplementary Table S2. [file 41598_2021_95705_MOESM3_ESM.docx]

**Supplementary Table 2 A: The common 65 DEGs for decidualization for all groups with E2P4 ± DHT post treatment.**

|  |  | **E2P4 *vs.* E2 _Ctrl** | | | **E2P4DHT *vs.* E2DHT_Ctrl** | | | **E2P4 *vs.* E2 _PCOS** | | | **E2P4DHT *vs.* E2DHT_PCOS** | | |
| --- | --- | --- | --- | --- | --- | --- | --- | --- | --- | --- | --- | --- | --- |
| Ensembl_ID | Gene name | logMean | LFC | FDR | logMean | LFC | FDR | logMean | LFC | FDR | logMean | LFC | FDR |
| ENSG00000157005 | SST | 3.19 | 7.45 | 0.000001652 | 2.95 | 7.48 | 0.000001016 | 3.03 | 6.25 | 0.00000426 | 3.14 | 6.81 | 9.029E-09 |
| ENSG00000123496 | IL13RA2 | 5.13 | 6.59 | 5.733E-13 | 5.50 | 7.08 | 1.707E-14 | 4.65 | 4.61 | 0.00007055 | 4.82 | 5.41 | 1.24E-09 |
| ENSG00000143839 | REN | 5.27 | 5.66 | 1.18E-15 | 5.74 | 5.08 | 5.336E-18 | 5.28 | 5.39 | 3.521E-27 | 5.25 | 5.82 | 1.768E-26 |
| ENSG00000099260 | PALMD | 5.11 | 2.75 | 0.01376 | 4.65 | 2.64 | 0.0001843 | 4.84 | 2.31 | 0.0002212 | 4.81 | 1.53 | 0.03654 |
| ENSG00000164761 | TNFRSF11B | 6.68 | 2.44 | 0.0004466 | 6.96 | 3.32 | 1.707E-14 | 6.89 | 2.37 | 0.00002025 | 7.00 | 2.62 | 0.000001297 |
| ENSG00000100473 | COCH | 5.33 | 2.34 | 0.0005802 | 5.64 | 2.68 | 0.001202 | 5.71 | 2.50 | 0.00002306 | 5.73 | 2.25 | 0.0001796 |
| ENSG00000149968 | MMP3 | 9.28 | 2.11 | 0.03511 | 9.27 | 2.69 | 0.0001232 | 9.22 | 1.88 | 0.01587 | 9.13 | 1.64 | 0.007376 |
| ENSG00000137868 | STRA6 | 5.47 | -0.95 | 0.03773 | 5.51 | -1.14 | 0.001141 | 5.50 | -1.07 | 0.03936 | 5.59 | -1.10 | 0.04623 |
| ENSG00000133466 | C1QTNF6 | 5.21 | -1.27 | 0.02123 | 5.10 | -1.11 | 0.02955 | 5.12 | -1.06 | 0.01545 | 5.12 | -1.21 | 0.04305 |
| ENSG00000145147 | SLIT2 | 8.31 | -1.47 | 0.01548 | 8.29 | -2.00 | 0.0000254 | 8.14 | -2.64 | 0.002405 | 7.90 | -1.93 | 0.01602 |
| ENSG00000213347 | MXD3 | 3.18 | -1.54 | 0.045 | 3.17 | -2.96 | 0.000000276 | 3.35 | -1.60 | 0.01371 | 3.10 | -1.83 | 0.001055 |
| ENSG00000113657 | DPYSL3 | 6.01 | -1.61 | 0.01048 | 5.76 | -1.06 | 0.008428 | 5.64 | -1.12 | 0.03324 | 5.58 | -1.17 | 0.00585 |
| ENSG00000099953 | MMP11 | 9.49 | -1.63 | 0.01793 | 9.51 | -1.57 | 0.008676 | 9.50 | -1.82 | 0.00001124 | 9.46 | -1.96 | 3.542E-09 |
| ENSG00000123080 | CDKN2C | 3.70 | -1.68 | 0.01602 | 3.75 | -2.29 | 0.0000787 | 3.76 | -1.88 | 0.01097 | 3.76 | -1.74 | 0.009443 |
| ENSG00000120802 | TMPO | 6.90 | -1.70 | 0.01415 | 7.04 | -1.99 | 0.00001136 | 6.78 | -1.72 | 0.009457 | 6.59 | -1.24 | 0.01812 |
| ENSG00000162063 | CCNF | 5.57 | -1.72 | 0.01245 | 5.66 | -1.78 | 0.0002104 | 5.51 | -1.98 | 0.0007289 | 5.45 | -1.22 | 0.006366 |
| ENSG00000092470 | WDR76 | 7.54 | -1.79 | 0.0005467 | 7.63 | -1.65 | 0.005582 | 7.55 | -1.61 | 0.00007135 | 7.50 | -1.26 | 0.008668 |
| ENSG00000164692 | COL1A2 | 9.49 | -1.81 | 0.00001667 | 9.49 | -1.55 | 0.00005937 | 9.40 | -1.59 | 1.002E-08 | 9.40 | -1.70 | 4.085E-08 |
| ENSG00000129195 | PIMREG | 4.19 | -1.81 | 0.01218 | 4.12 | -2.12 | 0.0002329 | 4.19 | -2.41 | 4.187E-07 | 4.40 | -2.80 | 0.0001685 |
| ENSG00000164104 | HMGB2 | 5.30 | -1.91 | 0.01887 | 5.13 | -1.91 | 0.009813 | 4.94 | -2.50 | 2.978E-09 | 5.26 | -2.09 | 0.000003699 |
| ENSG00000137563 | GGH | 6.22 | -1.92 | 0.007979 | 6.16 | -1.55 | 0.01753 | 6.15 | -2.02 | 5.193E-10 | 6.09 | -1.72 | 0.000002217 |
| ENSG00000013810 | TACC3 | 4.70 | -1.99 | 0.000219 | 4.63 | -2.17 | 1.938E-09 | 4.61 | -2.06 | 5.193E-10 | 4.63 | -2.05 | 1.91E-09 |
| ENSG00000163431 | LMOD1 | 4.65 | -2.02 | 0.00003277 | 4.61 | -1.47 | 0.009693 | 4.43 | -1.25 | 0.0007192 | 4.55 | -1.43 | 0.04623 |
| ENSG00000138131 | LOXL4 | 5.67 | -2.09 | 0.003776 | 5.76 | -2.21 | 0.00008086 | 6.12 | -2.58 | 0.00001475 | 6.09 | -2.30 | 0.0008623 |
| ENSG00000168077 | SCARA3 | 4.47 | -2.15 | 0.005879 | 4.25 | -2.19 | 0.0002072 | 4.49 | -1.75 | 0.00001091 | 4.56 | -2.07 | 2.458E-09 |
| ENSG00000155760 | FZD7 | 4.34 | -2.20 | 0.0003481 | 4.18 | -1.82 | 0.0004303 | 4.14 | -1.98 | 0.00004476 | 3.97 | -1.95 | 0.000002451 |
| ENSG00000089685 | BIRC5 | 6.36 | -2.21 | 0.002285 | 6.26 | -2.07 | 0.00005993 | 6.37 | -2.74 | 0.000003995 | 6.01 | -1.82 | 0.0001545 |
| ENSG00000101447 | FAM83D | 3.24 | -2.25 | 0.03751 | 3.50 | -2.22 | 0.001992 | 3.43 | -2.40 | 3.476E-08 | 3.28 | -2.16 | 0.001046 |
| ENSG00000137804 | NUSAP1 | 5.37 | -2.27 | 0.00952 | 4.97 | -2.13 | 0.04132 | 5.31 | -2.27 | 0.02508 | 5.05 | -2.67 | 0.0002944 |
| ENSG00000129009 | ISLR | 6.68 | -2.30 | 6.786E-07 | 6.99 | -2.03 | 0.01627 | 6.92 | -1.77 | 0.04877 | 7.06 | -2.43 | 0.001087 |
| ENSG00000182492 | BGN | 5.32 | -2.47 | 0.00002859 | 5.22 | -2.49 | 0.000001412 | 4.52 | -2.12 | 0.000001928 | 4.53 | -1.96 | 0.0007126 |
| ENSG00000166851 | PLK1 | 4.77 | -2.49 | 0.004813 | 4.35 | -2.67 | 0.001449 | 4.13 | -2.18 | 0.0008572 | 4.47 | -1.86 | 0.03011 |
| ENSG00000185745 | IFIT1 | 5.47 | -2.49 | 0.004374 | 5.22 | -2.12 | 0.004135 | 5.69 | -3.09 | 3.323E-08 | 5.24 | -2.25 | 0.00001598 |
| ENSG00000065911 | MTHFD2 | 6.42 | -2.58 | 0.00002803 | 6.47 | -2.08 | 0.0001803 | 6.04 | -1.78 | 0.00005097 | 6.08 | -1.56 | 0.005743 |
| ENSG00000091986 | CCDC80 | 6.58 | -2.65 | 0.0009756 | 6.62 | -3.03 | 7.401E-08 | 6.64 | -3.29 | 0.00003582 | 6.73 | -3.30 | 0.0002291 |
| ENSG00000134057 | CCNB1 | 5.23 | -2.68 | 0.007979 | 4.86 | -3.64 | 1.456E-08 | 4.90 | -3.81 | 1.128E-08 | 5.03 | -3.53 | 2.864E-10 |
| ENSG00000107562 | CXCL12 | 4.72 | -2.69 | 0.000219 | 4.78 | -2.75 | 0.006794 | 4.86 | -2.91 | 6.179E-08 | 4.86 | -3.22 | 2.899E-15 |
| ENSG00000108821 | COL1A1 | 11.32 | -2.70 | 6.041E-12 | 11.25 | -2.17 | 5.185E-09 | 11.13 | -2.27 | 1.225E-09 | 11.03 | -2.04 | 2.486E-08 |
| ENSG00000041982 | TNC | 5.38 | -2.70 | 0.01223 | 5.81 | -5.15 | 4.282E-15 | 4.64 | -2.68 | 0.000883 | 4.94 | -2.41 | 0.01602 |
| ENSG00000157456 | CCNB2 | 4.25 | -2.75 | 0.0000238 | 4.62 | -3.55 | 7.275E-08 | 4.74 | -3.87 | 1.061E-12 | 4.67 | -3.98 | 5.627E-13 |
| ENSG00000164611 | PTTG1 | 5.58 | -2.82 | 0.001353 | 5.09 | -2.47 | 0.02565 | 4.95 | -4.10 | 1.967E-14 | 4.93 | -2.33 | 0.006247 |
| ENSG00000186193 | SAPCD2 | 4.45 | -2.85 | 0.0003481 | 4.27 | -2.61 | 0.00001865 | 4.18 | -1.88 | 0.005114 | 4.10 | -1.62 | 0.0001813 |
| ENSG00000115414 | FN1 | 8.14 | -2.90 | 4.875E-13 | 8.16 | -2.75 | 4.953E-11 | 7.55 | -2.17 | 0.000007179 | 7.56 | -2.04 | 0.00002584 |
| ENSG00000004776 | HSPB6 | 5.63 | -3.01 | 7.458E-07 | 5.78 | -3.15 | 3.121E-18 | 4.88 | -2.68 | 0.00001047 | 4.93 | -2.57 | 0.00003086 |
| ENSG00000111341 | MGP | 6.51 | -3.02 | 0.002332 | 6.38 | -4.06 | 3.255E-08 | 5.94 | -3.18 | 0.000176 | 6.27 | -2.98 | 0.0009053 |
| ENSG00000107796 | ACTA2 | 7.58 | -3.09 | 9.437E-10 | 7.45 | -2.72 | 7.846E-13 | 7.06 | -1.86 | 0.0001072 | 6.97 | -1.96 | 0.00002745 |
| ENSG00000092621 | PHGDH | 5.68 | -3.09 | 1.407E-08 | 5.60 | -2.40 | 0.000006921 | 5.48 | -2.35 | 2.467E-09 | 5.58 | -2.74 | 2.864E-10 |
| ENSG00000166482 | MFAP4 | 7.42 | -3.13 | 0.00001596 | 7.24 | -3.22 | 6.294E-08 | 6.83 | -3.06 | 2.682E-12 | 6.80 | -3.39 | 1.047E-16 |
| ENSG00000187955 | COL14A1 | 5.36 | -3.19 | 3.692E-09 | 5.50 | -3.29 | 2.289E-07 | 5.92 | -3.25 | 4.264E-09 | 5.91 | -3.08 | 5.906E-09 |
| ENSG00000100162 | CENPM | 4.80 | -3.27 | 0.00105 | 4.01 | -2.36 | 0.001315 | 3.93 | -2.29 | 0.0002213 | 4.60 | -2.36 | 0.002901 |
| ENSG00000135069 | PSAT1 | 5.20 | -3.30 | 7.702E-08 | 5.46 | -3.03 | 7.275E-08 | 5.07 | -1.96 | 0.000214 | 5.03 | -2.10 | 0.0009942 |
| ENSG00000164099 | PRSS12 | 5.34 | -3.52 | 3.654E-10 | 5.19 | -2.56 | 0.00009588 | 5.37 | -3.42 | 0.004565 | 4.93 | -2.38 | 0.04217 |
| ENSG00000133110 | POSTN | 6.18 | -3.67 | 0.00002808 | 6.39 | -3.51 | 0.0006498 | 5.25 | -4.90 | 0.0000106 | 4.89 | -4.20 | 0.000073 |
| ENSG00000131747 | TOP2A | 4.72 | -3.71 | 0.00001135 | 4.70 | -3.78 | 8.961E-08 | 4.06 | -2.36 | 0.00007662 | 4.52 | -3.44 | 1.157E-12 |
| ENSG00000106819 | ASPN | 5.75 | -3.71 | 0.000582 | 5.58 | -3.35 | 0.004326 | 4.79 | -3.05 | 0.000883 | 5.21 | -2.33 | 0.028 |
| ENSG00000171848 | RRM2 | 6.46 | -3.88 | 0.00002859 | 5.95 | -3.25 | 1.735E-09 | 6.14 | -3.53 | 5.226E-14 | 6.07 | -3.38 | 1.067E-12 |
| ENSG00000143228 | NUF2 | 4.20 | -3.97 | 0.00215 | 3.81 | -4.10 | 0.0002329 | 3.98 | -4.79 | 5.524E-07 | 3.41 | -4.24 | 0.0003222 |
| ENSG00000164109 | MAD2L1 | 5.58 | -4.01 | 0.000006449 | 4.64 | -2.09 | 0.04989 | 4.57 | -2.23 | 0.01989 | 4.86 | -3.11 | 8.635E-07 |
| ENSG00000139734 | DIAPH3 | 7.53 | -4.40 | 2.133E-10 | 7.13 | -3.09 | 0.00005437 | 7.06 | -5.46 | 1.439E-18 | 6.99 | -4.16 | 4.654E-20 |
| ENSG00000175063 | UBE2C | 4.28 | -4.58 | 9.899E-11 | 4.29 | -4.83 | 2.655E-14 | 4.16 | -5.06 | 1.646E-30 | 3.99 | -4.33 | 3.689E-15 |
| ENSG00000161888 | SPC24 | 7.32 | -4.78 | 1.357E-26 | 7.43 | -4.94 | 5.473E-12 | 7.12 | -4.13 | 7.051E-23 | 7.06 | -4.15 | 4.14E-23 |
| ENSG00000168078 | PBK | 4.50 | -5.84 | 1.569E-11 | 4.18 | -5.26 | 2.523E-09 | 3.78 | -5.20 | 5.034E-15 | 3.29 | -3.57 | 1.118E-07 |
| ENSG00000117399 | CDC20 | 4.42 | -6.07 | 8.977E-15 | 4.56 | -5.18 | 7.325E-10 | 4.40 | -5.27 | 1.242E-17 | 4.19 | -4.46 | 6.719E-17 |
| ENSG00000183856 | IQGAP3 | 8.37 | -6.48 | 2.037E-41 | 8.10 | -6.57 | 1.894E-35 | 8.16 | -7.02 | 1.867E-93 | 7.99 | -5.56 | 1.722E-23 |
| ENSG00000100526 | CDKN3 | 6.04 | -6.63 | 3.749E-15 | 4.84 | -3.47 | 0.006777 | 5.44 | -2.47 | 0.04108 | 5.88 | -4.44 | 0.01016 |

**Supplementary Table 2 B: 19 common DEGs in E2P4 vs. E2 comparison in eSC_Ctrl_ and eSC_PCOS_**

|  |  | **E2P4 *vs.* E2_Ctrl** | | | **E2P4 *vs.* E2_PCOS** | | |
| --- | --- | --- | --- | --- | --- | --- | --- |
| Ensembl_ID | Gene name | logMean | LFC | FDR | logMean | LFC | FDR |
| ENSG00000188959 | C9orf152 | 2.31 | 5.56 | 0.001808 | 2.34 | 2.93 | 0.02987 |
| ENSG00000185040 | SPDYE16 | 2.81 | 3.89 | 0.02925 | 3.31 | 1.49 | 0.03187 |
| ENSG00000113389 | NPR3 | 5.20 | 3.33 | 0.00461 | 4.33 | 2.79 | 0.02955 |
| ENSG00000122025 | FLT3 | 2.58 | 3.21 | 0.01491 | 2.45 | 3.35 | 0.001405 |
| ENSG00000156510 | HKDC1 | 2.55 | 2.56 | 0.03187 | 3.78 | 4.51 | 0.002745 |
| ENSG00000033627 | ATP6V0A1 | 5.49 | 1.12 | 0.0318 | 5.31 | 0.87 | 0.04919 |
| ENSG00000119865 | CNRIP1 | 5.68 | -1.20 | 0.01854 | 5.61 | -1.28 | 0.0002212 |
| ENSG00000087116 | ADAMTS2 | 5.66 | -1.30 | 0.004888 | 5.58 | -1.29 | 0.0005317 |
| ENSG00000183010 | PYCR1 | 4.60 | -1.39 | 0.00322 | 4.66 | -1.43 | 0.007075 |
| ENSG00000100889 | PCK2 | 4.40 | -1.42 | 0.00359 | 4.58 | -1.72 | 0.00001409 |
| ENSG00000182054 | IDH2 | 5.70 | -1.47 | 0.01381 | 5.68 | -1.26 | 0.006187 |
| ENSG00000178573 | MAF | 7.43 | -1.52 | 0.01632 | 7.22 | -1.76 | 0.003017 |
| ENSG00000163017 | ACTG2 | 3.74 | -1.97 | 0.0157 | 4.52 | -2.29 | 0.004653 |
| ENSG00000078177 | N4BP2 | 6.17 | -2.40 | 0.03394 | 5.99 | -3.05 | 0.0008805 |
| ENSG00000071282 | LMCD1 | 5.43 | -2.66 | 0.000582 | 5.04 | -1.92 | 0.02161 |
| ENSG00000119922 | IFIT2 | 3.90 | -3.19 | 0.0008157 | 4.23 | -3.71 | 0.005948 |
| ENSG00000163145 | C1QTNF7 | 5.73 | -3.88 | 0.00005462 | 5.10 | -2.17 | 0.006982 |
| ENSG00000106483 | SFRP4 | 4.95 | -4.04 | 0.00003436 | 4.21 | -3.03 | 0.007675 |
| ENSG00000122952 | ZWINT | 4.30 | -4.68 | 0.0003097 | 3.77 | -2.97 | 0.004572 |

**Supplementary Table 2 C: 18 common DEGs in E2P4DHT vs. E2DHT comparison in eSC_Ctrl_ and eSC_PCOS_**

|  |  | **E2P4DHT *vs.* E2DHT_Ctrl** | | | **E2P4DHT *vs.* E2DHT_PCOS** | | |
| --- | --- | --- | --- | --- | --- | --- | --- |
| Ensembl_ID | Gene name | logMean | LFC | FDR | logMean | LFC | FDR |
| ENSG00000273079 | GRIN2B | 5.58 | 5.10 | 0.0412 | 3.45 | 2.29 | 0.04623 |
| ENSG00000117600 | PLPPR4 | 5.28 | 2.76 | 0.01009 | 5.50 | 1.68 | 0.02377 |
| ENSG00000151148 | UBE3B | 5.81 | 1.94 | 0.006838 | 5.52 | 1.62 | 0.009541 |
| ENSG00000169908 | TM4SF1 | 4.39 | 1.93 | 0.01929 | 4.41 | 2.24 | 0.03432 |
| ENSG00000113739 | STC2 | 6.14 | 1.22 | 0.04958 | 5.89 | 1.13 | 0.007491 |
| ENSG00000130513 | GDF15 | 7.22 | 1.15 | 0.04457 | 7.19 | 1.17 | 0.02496 |
| ENSG00000070669 | ASNS | 4.63 | -1.23 | 0.01133 | 4.40 | -1.04 | 0.004082 |
| ENSG00000181751 | C5orf30 | 4.75 | -1.32 | 0.001688 | 4.48 | -1.35 | 0.01956 |
| ENSG00000163923 | RPL39L | 4.81 | -1.40 | 0.01954 | 5.00 | -1.72 | 0.000003655 |
| ENSG00000152377 | SPOCK1 | 6.53 | -1.59 | 0.001937 | 6.22 | -1.49 | 0.005138 |
| ENSG00000106462 | EZH2 | 4.92 | -2.02 | 0.03235 | 4.76 | -1.83 | 0.006515 |
| ENSG00000198901 | PRC1 | 4.10 | -2.09 | 0.02935 | 4.09 | -1.95 | 0.00005866 |
| ENSG00000123096 | SSPN | 5.01 | -2.12 | 0.00000018 | 4.35 | -1.79 | 0.03023 |
| ENSG00000123485 | HJURP | 3.98 | -2.22 | 0.001398 | 3.82 | -1.70 | 0.0251 |
| ENSG00000272419 | LINC01145 | 4.74 | -2.78 | 0.002898 | 4.48 | -2.24 | 0.04479 |
| ENSG00000214274 | ANG | 2.97 | -2.80 | 0.0002982 | 3.32 | -3.73 | 0.0007282 |
| ENSG00000166451 | CENPN | 4.97 | -3.18 | 7.275E-08 | 5.23 | -3.57 | 0.000008581 |
| ENSG00000198121 | LPAR1 | 4.88 | -3.25 | 0.000838 | 4.47 | -3.49 | 0.00008027 |

DEG, differentially expressed genes; LFC, log fold change; FDR, false discovery rate; *versus, vs.*
