# Supplementary material for: Decidualized endometrial stromal cells present with altered androgen response in PCOS
Source: Sci Rep. 2021 Aug 11;11:16287. doi: 10.1038/s41598-021-95705-0 (PMC8357821; doi:10.1038/s41598-021-95705-0)
Supplement: Supplementary file 5 — Supplementary Table S4. [file 41598_2021_95705_MOESM5_ESM.docx]

**Supplemental Table 4: Differentially expressed genes (DEGs) filtered by Independent Hypothesis Weighing Bonferroni (IHW-BON)**

| **A) 13 out of 140 DEGs in E2P4 *vs.* E2 unique for eSC_Ctrl_** | | | | | |
| --- | --- | --- | --- | --- | --- |
| **Ensembl_Gene_ID** | **Gene name** | **LFC** | **P-value** | **FDR** | **IHW-BON** |
| ENSG00000233210 | GPANK1 | 7.404 | 2.0E-08 | 1.5E-05 | 0.00072 |
| ENSG00000131470 | PSMC3IP | 4.646 | 6.9E-15 | 1.6E-11 | 0.00000 |
| ENSG00000165480 | SKA3 | 4.458 | 5.3E-07 | 2.4E-04 | 0.00699 |
| ENSG00000082482 | KCNK2 | 4.380 | 6.1E-07 | 2.6E-04 | 0.00767 |
| ENSG00000147180 | ZNF711 | 4.347 | 6.9E-07 | 2.9E-04 | 0.00913 |
| ENSG00000162595 | DIRAS3 | 3.640 | 7.5E-07 | 3.1E-04 | 0.00994 |
| ENSG00000188985 | DHFRP1 | 3.589 | 1.1E-06 | 3.9E-04 | 0.03781 |
| ENSG00000141448 | GATA6 | 2.839 | 3.4E-07 | 1.6E-04 | 0.02436 |
| ENSG00000081320 | STK17B | 1.581 | 9.3E-08 | 4.7E-05 | 0.00118 |
| ENSG00000158445 | KCNB1 | -3.263 | 7.5E-07 | 3.1E-04 | 0.01321 |
| ENSG00000143614 | GATAD2B | -4.234 | 8.9E-07 | 3.5E-04 | 0.03232 |
| ENSG00000188659 | SAXO2 | -5.325 | 4.2E-09 | 3.6E-06 | 0.00003 |
| ENSG00000175449 | RFESD | -5.706 | 5.6E-08 | 3.0E-05 | 0.00074 |
| **B) 19 out of 251 DEGs in E2P4 *vs.* E2 unique for eSC_PCOS_** | | | | | |
| **Ensembl_Gene_ID** | **Gene name** | **LFC** | **P-value** | **FDR** | **IHW-BON** |
| ENSG00000230092 | AL669831.4 | 6.025 | 4.6E-07 | 0.00013 | 0.00669 |
| ENSG00000031691 | CENPQ | 4.474 | 6.7E-10 | 0.00000 | 0.00002 |
| ENSG00000070718 | AP3M2 | 3.990 | 7.4E-07 | 0.00019 | 0.01796 |
| ENSG00000118849 | RARRES1 | 3.815 | 2.6E-06 | 0.00050 | 0.02474 |
| ENSG00000122877 | EGR2 | 2.141 | 8.2E-07 | 0.00020 | 0.01193 |
| ENSG00000135338 | LCA5 | 1.974 | 1.5E-06 | 0.00033 | 0.02560 |
| ENSG00000162746 | FCRLB | 1.915 | 9.0E-07 | 0.00022 | 0.01539 |
| ENSG00000164687 | FABP5 | 1.756 | 1.1E-06 | 0.00025 | 0.01628 |
| ENSG00000078674 | PCM1 | 1.664 | 1.9E-06 | 0.00040 | 0.02828 |
| ENSG00000131016 | AKAP12 | 1.239 | 1.7E-06 | 0.00036 | 0.02489 |
| ENSG00000125826 | RBCK1 | 0.967 | 6.8E-07 | 0.00018 | 0.01006 |
| ENSG00000059804 | SLC2A3 | -1.482 | 1.0E-06 | 0.00023 | 0.01497 |
| ENSG00000121966 | CXCR4 | -1.920 | 1.3E-06 | 0.00029 | 0.02230 |
| ENSG00000164161 | HHIP | -2.081 | 7.5E-07 | 0.00019 | 0.01094 |
| ENSG00000185736 | ADARB2 | -2.147 | 1.6E-06 | 0.00036 | 0.01808 |
| ENSG00000168878 | SFTPB | -3.422 | 6.3E-11 | 0.00000 | 0.00000 |
| ENSG00000237289 | CKMT1B | -4.177 | 4.3E-07 | 0.00012 | 0.03105 |
| ENSG00000095574 | IKZF5 | -4.248 | 2.0E-06 | 0.00041 | 0.04780 |
| ENSG00000085563 | ABCB1 | -4.760 | 1.3E-08 | 0.00001 | 0.00022 |
| **C) 13 out of 136 DEGs in E2P4DHT *vs.* E2DHT unique for eSC_Ctrl_** | | | | | |
| **Ensembl_Gene_ID** | **Gene name** | **LFC** | **P-value** | **FDR** | **IHW-BON** |
| ENSG00000169429 | CXCL8 | 7.635 | 7.9E-11 | 6.5E-08 | 2.5E-06 |
| ENSG00000102543 | CDADC1 | 6.346 | 9.4E-10 | 5.0E-07 | 6.5E-05 |
| ENSG00000125703 | ATG4C | 4.327 | 3.3E-08 | 1.3E-05 | 4.1E-04 |
| ENSG00000162892 | IL24 | 3.848 | 6.2E-09 | 2.8E-06 | 7.6E-05 |
| ENSG00000138050 | THUMPD2 | 3.623 | 3.8E-07 | 1.2E-04 | 4.7E-03 |
| ENSG00000185973 | TMLHE | 3.504 | 1.8E-07 | 6.1E-05 | 7.1E-03 |
| ENSG00000166670 | MMP10 | 2.793 | 1.4E-07 | 5.2E-05 | 1.7E-03 |
| ENSG00000139508 | SLC46A3 | 2.730 | 1.3E-06 | 3.2E-04 | 2.4E-02 |
| ENSG00000108932 | SLC16A6 | 2.277 | 5.0E-07 | 1.5E-04 | 6.4E-03 |
| ENSG00000168961 | LGALS9 | -2.338 | 7.2E-07 | 2.0E-04 | 8.9E-03 |
| ENSG00000135976 | ANKRD36 | -2.364 | 3.3E-06 | 6.9E-04 | 4.0E-02 |
| ENSG00000104147 | OIP5 | -2.370 | 6.1E-07 | 1.8E-04 | 2.5E-02 |
| ENSG00000117586 | TNFSF4 | -2.536 | 1.4E-06 | 3.3E-04 | 2.5E-02 |
| **D) 17 out of 314 DEGs in E2P4DHT *vs.* E2DHT unique for eSC_PCOS_** | | | | | |
| **Ensembl_Gene_ID** | **Gene name** | **LFC** | **P-value** | **FDR** | **IHW-BON** |
| ENSG00000102174 | PHEX | 3.814 | 6.1E-07 | 0.00015 | 0.02203 |
| ENSG00000197121 | PGAP1 | 3.515 | 1.4E-07 | 0.00004 | 0.00182 |
| ENSG00000006459 | KDM7A | 3.426 | 2.4E-06 | 0.00045 | 0.03286 |
| ENSG00000108306 | FBXL20 | 3.340 | 5.9E-08 | 0.00002 | 0.00086 |
| ENSG00000152932 | RAB3C | 3.192 | 2.6E-07 | 0.00007 | 0.01093 |
| ENSG00000183474 | GTF2H2C | 3.057 | 6.9E-10 | 0.00000 | 0.00001 |
| ENSG00000276203 | ANKRD20A3 | 2.559 | 9.6E-08 | 0.00003 | 0.00127 |
| ENSG00000258818 | RNASE4 | -1.823 | 2.2E-07 | 0.00006 | 0.00244 |
| ENSG00000028528 | SNX1 | -1.829 | 4.0E-06 | 0.00071 | 0.04965 |
| ENSG00000250722 | SELENOP | -1.833 | 1.0E-07 | 0.00003 | 0.00148 |
| ENSG00000113248 | PCDHB15 | -1.880 | 2.1E-07 | 0.00006 | 0.00801 |
| ENSG00000117228 | GBP1 | -2.124 | 7.5E-08 | 0.00003 | 0.00109 |
| ENSG00000142552 | RCN3 | -2.372 | 7.5E-07 | 0.00017 | 0.00919 |
| ENSG00000114439 | BBX | -2.497 | 3.9E-07 | 0.00010 | 0.00583 |
| ENSG00000275214 | IFI27 | -3.129 | 4.6E-08 | 0.00002 | 0.00061 |
| ENSG00000165092 | ALDH1A1 | -3.204 | 2.5E-06 | 0.00046 | 0.03650 |
| ENSG00000155754 | C2CD6 | -4.831 | 3.6E-06 | 0.00064 | 0.04782 |

LFC, log fold change; FDR, false discovery rate; *versus, vs.*
