# Supplementary figures and images for: Decidualized endometrial stromal cells present with altered androgen response in PCOS
Source: Sci Rep. 2021 Aug 11;11:16287. doi: 10.1038/s41598-021-95705-0 (PMC8357821; doi:10.1038/s41598-021-95705-0)

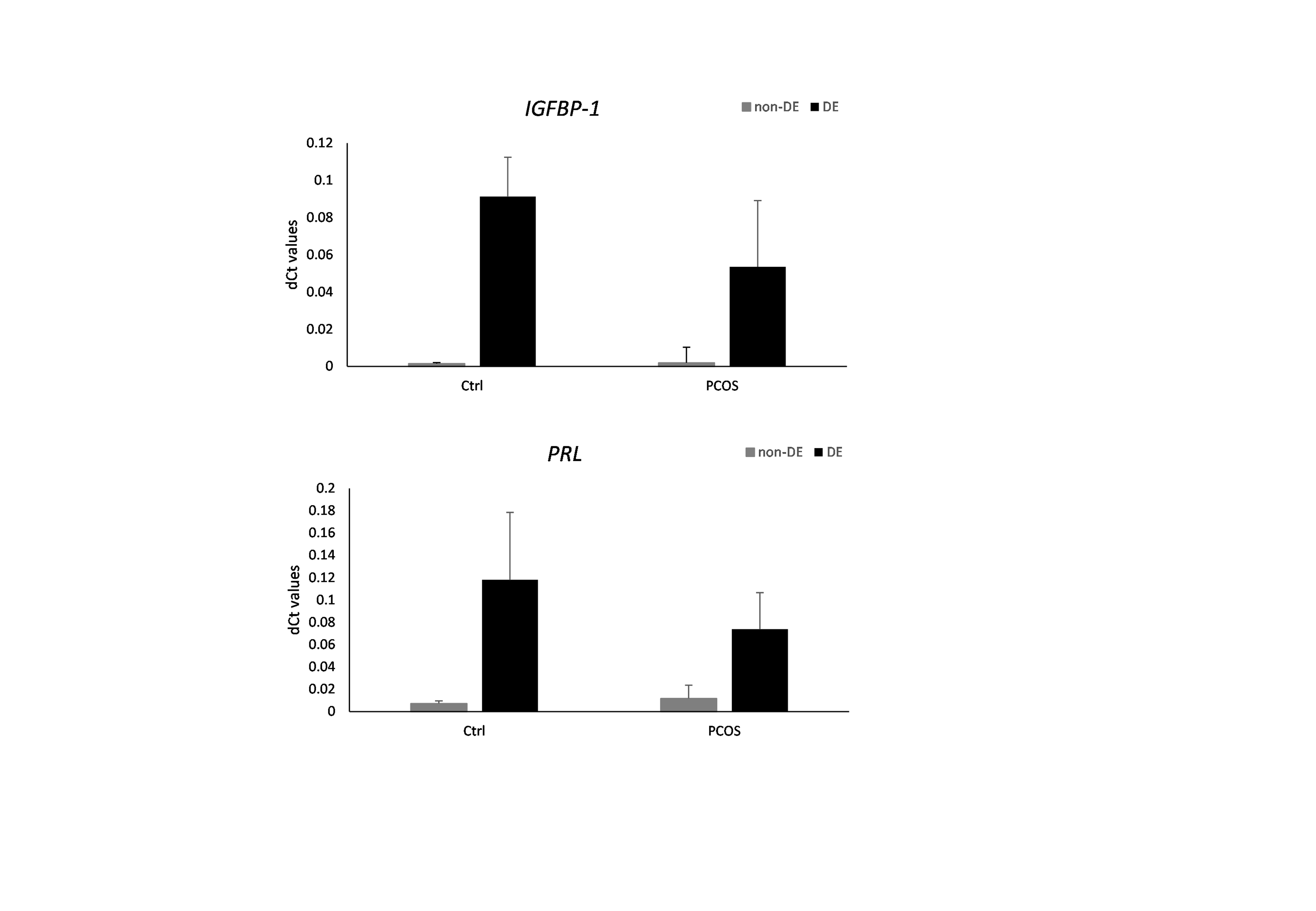

Supplement: Supplementary file 8 — Supplementary Figure 1. [file 41598_2021_95705_MOESM8_ESM.jpg]
