# Supplementary material for: Decidualized endometrial stromal cells present with altered androgen response in PCOS
Source: Sci Rep. 2021 Aug 11;11:16287. doi: 10.1038/s41598-021-95705-0 (PMC8357821; doi:10.1038/s41598-021-95705-0)
Supplement: Supplementary file 9 — Supplementary Captions. [file 41598_2021_95705_MOESM9_ESM.docx]

**Supplemental Figure 1: Decidualization confirmation by reverse transcription quantitative polymerase chain reaction (RT-qPCR).**

Decidualization classical markers, IGFBP-1 and PRL response were assessed from all samples (eSC_Ctrl_, eSC_PCOS_, n=6). All the decidualized eSCs (DE, black; 0.5 mM 8-Br-cAMP; diluted in DMSO for 96hrs) presented with increased IGFBP-1 and PRL compared to non-decidualized (non-DE, grey; 0.1% DMSO, vehicle for 96hrs). There was no significant difference between Ctrl and PCOS. *TBP* and *GAPDH* were used as reference genes.

**Supplemental Table S1**: **List of all differentially expressed genes (DEGs, FDR <0.05) induced by hormone post-treatment in PCOS and Ctrl.**

A) DEGs listed treatment-wise in PCOS *vs.* Ctrl. B) DEGs lists of treatments E2P4 *vs.* E2 and E2P4DHT *vs.* E2DHT in eSC_Ctrl_. C) DEGs lists of treatments E2P4 *vs.* E2 and E2P4DHT *vs.* E2DHT in eSC_PCOS_.

**Supplemental Table S2:** **Common differentially expressed genes (DEGs) for decidualization followed by steroid post-treatments.**

A) The common 65 DEGs for decidualization for all groups with E2P4 ± DHT post treatment. B) 19 common DEGs in E2P4 *vs.* E2 in eSC_Ctrl_ and eSC_PCOS_. C) 18 common DEGs in E2P4DHT *vs.* E2DHT in eSC_Ctrl_ and eSC_PCOS_.

**Supplemental Table S3: List of unique differentially expressed genes (DEGs, FDR <0.05) in eSC_Ctrl_** **and eSC_PCOS_ with a steroid hormone post-treatment.**

A) List of 140 DEGs uniquely expressed in E2P4 *vs.* E2 post-treated eSC_Ctrl_. B) List of 136 DEGs uniquely expressed in E2P4DHT *vs.* E2DHT post-treated eSC_Ctrl_. C) List of 251 DEGs uniquely expressed in E2P4 *vs.* E2 post-treated eSC_PCOS_. D) List of 314 DEGs uniquely expressed in E2P4DHT *vs.* E2DHT post-treated eSC_PCOS_.

**Supplemental Table S4: Differentially expressed genes (DEGs) filtered by Independent Hypothesis Weighing Bonferroni (IHW-BON).**

A) 13 out of 140 DEGs in E2P4 *vs.* E2 comparison unique for eSC_Ctrl_. B) 19 out of 251 DEGs in E2P4 *vs.* E2 unique for eSC_PCOS_. C) 13 out of 136 DEGs in E2P4DHT *vs.* E2DHT unique for eSC_Ctrl_ D) 17 out of 314 DEGs in E2P4DHT *vs.* E2DHT unique for eSC_PCOS_.

**Supplemental Table S5**: **List of Reactome and Gene Ontology (GO) data.**

A pathway enrichment analysis for A) 140 DEGs uniquely found in E2P4 *vs.* E2 post-treated eSC_Ctrl_; B) for 136 DEGs uniquely found in E2P4DHT *vs.* E2DHT post-treated eSC_Ctrl_; C) for 251 DEGs uniquely found in E2P4 *vs.* E2 post-treated eSC_PCOS_; D) for 314 DEGs uniquely found in E2P4DHT *vs.* E2DHT post-treated eSC_PCOS_; E) for 19 DEGs common for E2P4 *vs.* E2 post-treated eSC_Ctrl_ and eSC_PCOS_; F) for 18 DEGs common for E2P4DHT *vs.* E2DHT post-treated eSC_Ctrl_ and eSC_PCOS_; G) for 65 DEGs common in decidualization with E2P4 ± DHT *vs.* E2 ± DHT post-treatment in all groups.

**Supplemental Table S6: Primers used in reverse transcription quantitative polymerase chain reaction (RT-qPCR)** for validation of sequencing data and decidualization confirmation study.
